# Transcriptomic analyses of rice (*Oryza sativa*) genes and non-coding RNAs under nitrogen starvation using multiple omics technologies

**DOI:** 10.1186/s12864-018-4897-1

**Published:** 2018-07-13

**Authors:** Sang-Yoon Shin, Jin Seo Jeong, Jae Yun Lim, Taewook Kim, June Hyun Park, Ju-Kon Kim, Chanseok Shin

**Affiliations:** 1 Department of Agricultural Biotechnology, Seoul National University, Seoul, 08826 Republic of Korea; 2 Interdisciplinary Program in Agricultural Genomics, Seoul National University, Seoul, 08826 Republic of Korea; 3 Graduate School of International Agricultural Technology and Crop Biotechnology Institute/GreenBio Science & Technology, Seoul National University, Pyeongchang, 25354 Republic of Korea; 4 Research Institute of Agriculture and Life Sciences, and Plant Genomics and Breeding Institute, Seoul National University, Seoul, 08826 Republic of Korea; 5 Present address: Laboratory of Plant Molecular Biology, Rockefeller University, 1230 York Avenue, New York, NY 10065 USA

**Keywords:** Long non-coding RNA, microRNA, NGS, Nitrogen starvation, *Oryza sativa*, Transcriptome, Poly A-primed sequencing

## Abstract

**Background:**

Nitrogen (N) is a key macronutrient essential for plant growth, and its availability has a strong influence on crop development. The application of synthetic N fertilizers on crops has increased substantially in recent decades; however, the applied N is not fully utilized due to the low N use efficiency of crops. To overcome this limitation, it is important to understand the genome-wide responses and functions of key genes and potential regulatory factors in N metabolism.

**Results:**

Here, we characterized changes in the rice (*Oryza sativa*) transcriptome, including genes, newly identified putative long non-coding RNAs (lncRNAs), and microRNAs (miRNAs) and their target mRNAs in response to N starvation using four different transcriptome approaches. Analysis of rice genes involved in N metabolism and/or transport using strand-specific RNA-Seq identified 2588 novel putative lncRNA encoding loci. Analysis of previously published RNA-Seq datasets revealed a group of N starvation-responsive lncRNAs showing differential expression under other abiotic stress conditions. Poly A-primed sequencing (2P-Seq) revealed alternatively polyadenylated isoforms of N starvation-responsive lncRNAs and provided precise 3′ end information on the transcript models of these lncRNAs. Analysis of small RNA-Seq data identified N starvation-responsive miRNAs and down-regulation of miR169 family members, causing de-repression of NF-YA, as confirmed by strand-specific RNA-Seq and qRT-PCR. Moreover, we profiled the N starvation-responsive down-regulation of root-specific miRNA, osa-miR444a.4-3p, and Degradome sequencing confirmed *MADS25* as a novel target gene.

**Conclusions:**

In this study, we used a combination of multiple RNA-Seq analyses to extensively profile the expression of genes, newly identified lncRNAs, and microRNAs in N-starved rice roots and shoots. Data generated in this study provide an in-depth understanding of the regulatory pathways modulated by N starvation-responsive miRNAs. The results of comprehensive, large-scale data analysis provide valuable information on multiple aspects of the rice transcriptome, which may be useful in understanding the responses of rice plants to changes in the N supply status of soil.

**Electronic supplementary material:**

The online version of this article (10.1186/s12864-018-4897-1) contains supplementary material, which is available to authorized users.

## Background

Nitrogen (N) is a key macronutrient for plants and has a strong influence on crop development and productivity. To increase crop yield, the application of synthetic N fertilizers to crops has increased substantially in recent decades. However, plants utilize less than half of the applied N because of low N use efficiency (NUE) and uptake saturation [1]. The N fertilizers remaining in the soil poses several environmental problems, such as eutrophication. Moreover, increased total costs for N fertilizers lead to increases of product prices and a reduction in the farmer’s profitability. Therefore, improving crop NUE while maintaining crop productivity has several economic and environmental benefits.

Transgenic plants with improved NUE have been developed in which expression of protein-coding genes involved in N uptake, assimilation, and transport have been modulated by genetic engineering [2]. Overexpression of nitrate transporters [3–6] or ammonium transporters [7–10] led to enhanced N source uptake ability and increased nitrate and ammonium contents in transgenic plants. Overexpression of N assimilation enzymes, including alanine aminotransferase (AlaAT) [11, 12] and glutamine synthetase (GS) increased total N content and plant dry biomass and produced yield increases. In parallel with these genetic approaches, a number of N-responsive genes were identified using high-throughput analysis tools such as microarrays and next-generation sequencing (NGS) platforms [13–16]. In these studies, the expression profiles of genes have been examined under N-deficient conditions, and novel candidate genes for improving the NUE of crop plants via genetic engineering have been identified. Of the identified N-responsive genes, overexpression of *OsENOD93–1* (a rice early nodulin gene induced by N starvation) stimulated increases in shoot dry biomass and improvements in seed yield [13].

Recent studies of regulatory non-coding RNAs (ncRNAs), including microRNAs (miRNAs) and long non-coding RNAs (lncRNAs), identified their functional importance in many biological phenomena in plants including processes related to crop agricultural traits [17, 18]. Several large-scale investigations and functional studies of various stress-responsive ncRNAs in crops and other plants suggested that these regulatory ncRNAs had significant effects on physiological responses through regulation of gene [19–28]. Modulating expression of these regulatory ncRNAs could lead to improvements in some important agricultural traits such as productivity, male sterility, nutrient homeostasis, and floral organogenesis [19, 21, 29, 30]. Because of their functional significance and potential impact on agricultural traits, researchers are targeting ncRNAs as a novel resource for crop improvement [17, 31–33]. Researchers showed that changes in N supply status altered the expression of multiple miRNAs [34–38] and lncRNAs [25, 26] in several plant species. Many N-responsive miRNAs in plants were found to be involved in N metabolism [24, 36, 39], reprogramming root development [40], anthocyanin biosynthesis [41], and homeostasis of other nutrients [37]. However, while species-wide studies on plant miRNAs have been performed, no studies examining the involvement of lncRNAs in regulating N responses or metabolism in plants have been reported, with the exception of research in *Populus tomentosa* [25].

Many previous studies investigated the rice transcriptome in N-starved conditions using large-scale datasets to identify N-responsive genes and miRNAs [13–16, 34]. However, these studies were limited to a single type of RNA molecules, such as mRNAs or small RNAs, and did not provide an overview of the transcriptome-wide responses to changes in N availability. In this study, we combined multiple RNA-Seq analyses to assess multiple aspects of transcriptomes in N-starved rice, and to provide detailed information on transcriptome-wide changes in response to N availability. We profiled and analyzed diverse aspects of the rice transcriptome including genes, putative lncRNAs, and miRNAs and their target mRNAs, using four different types of RNA-Seq methods in N-starved rice samples prepared over a time course. These large-scale datasets analyzed in this study revealed N-responsive characteristics and expanded our knowledge of regulatory activities in N-starved rice, and provided insights towards understanding the molecular mechanisms underpinning modulation of N homeostasis in rice.

## Results

### Analysis of N-starved rice shoots and roots via various transcriptomic approaches

**Fig. 1:**
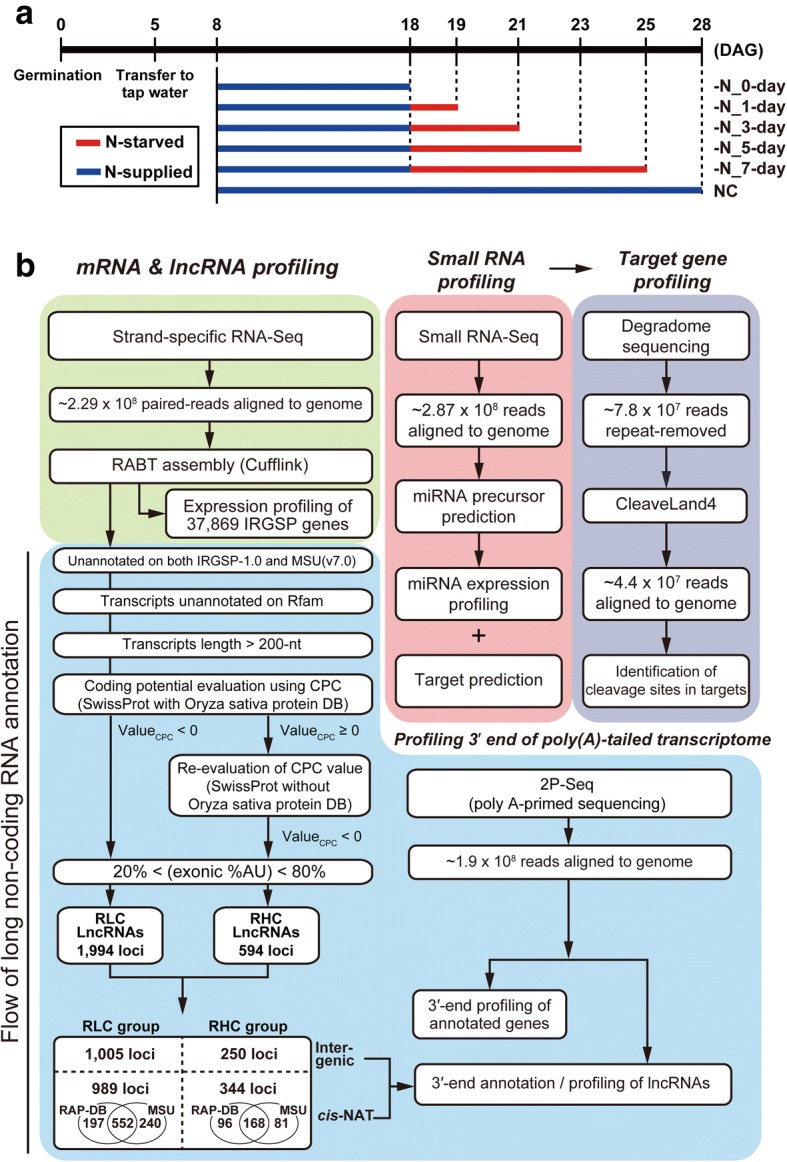
Sample preparation and omics analysis pipeline for nitrogen (N)-starved rice. **a** Experimental scheme for N-starved (-N) rice sample preparation. DAG, days after germination; NC, normal growth condition. **b** Integrated analysis pipeline for the identification and profiling of annotated rice genes, putative long non-coding RNAs, and microRNAs and their target genes, in N-starved rice samples

Four transcriptomic experimental tools were used to investigate the changes occurring in N-starved rice over a time course: strand-specific RNA-Seq, small RNA-Seq, 2P-Seq (poly A-primed sequencing), and Degradome sequencing (Fig. 1). N-starved samples were examined along with samples of 4-week-old rice plants grown under normal conditions (-N_0d) (Fig. 1a). All samples were analyzed by strand-specific RNA-Seq and small RNA-Seq. 2P-Seq and Degradome sequencing were applied to selected samples based on the analysis of the strand-specific RNA-Seq and small RNA-Seq datasets (Fig. 1b, Additional file 1: Figure. S1).

Approximately 513 million 101-bp paired-end reads generated from 12 strand-specific RNA-Seq datasets were mapped onto the rice genome (IRGSP-1.0). These reads were used for expression profiling of IRGSP-annotated genes (RAP-DB), and identifying previously unannotated putative lncRNAs based on the reference annotation-based transcript (RABT) assembly using Cufflinks [42]. In addition, 117 million reads from three 2P-Seq analyses were examined to characterize the 3′ ends of IRGSP-annotated genes and novel putative lncRNAs. Together with strand-specific RNA-Seq reads, strand-specific 2P-Seq reads were helpful to confirm the expression of *cis*-natural antisense transcripts (*cis*-NATs). For rice miRNA profiling, 287 million genome-aligned small RNA-Seq reads were analyzed. After expression profiling of previously annotated rice miRNAs, Degradome data analysis was performed in 7 day N-starved roots to investigate targets of N starvation-responsive miRNAs. Altogether, these omics-based high-throughput analyses provided a spatio-temporal characterization of the rice transcriptome in response to N starvation.

### Gene expression profiling of genes involved in N source transport and assimilation using strand-specific RNA-Seq data

**Fig. 2:**
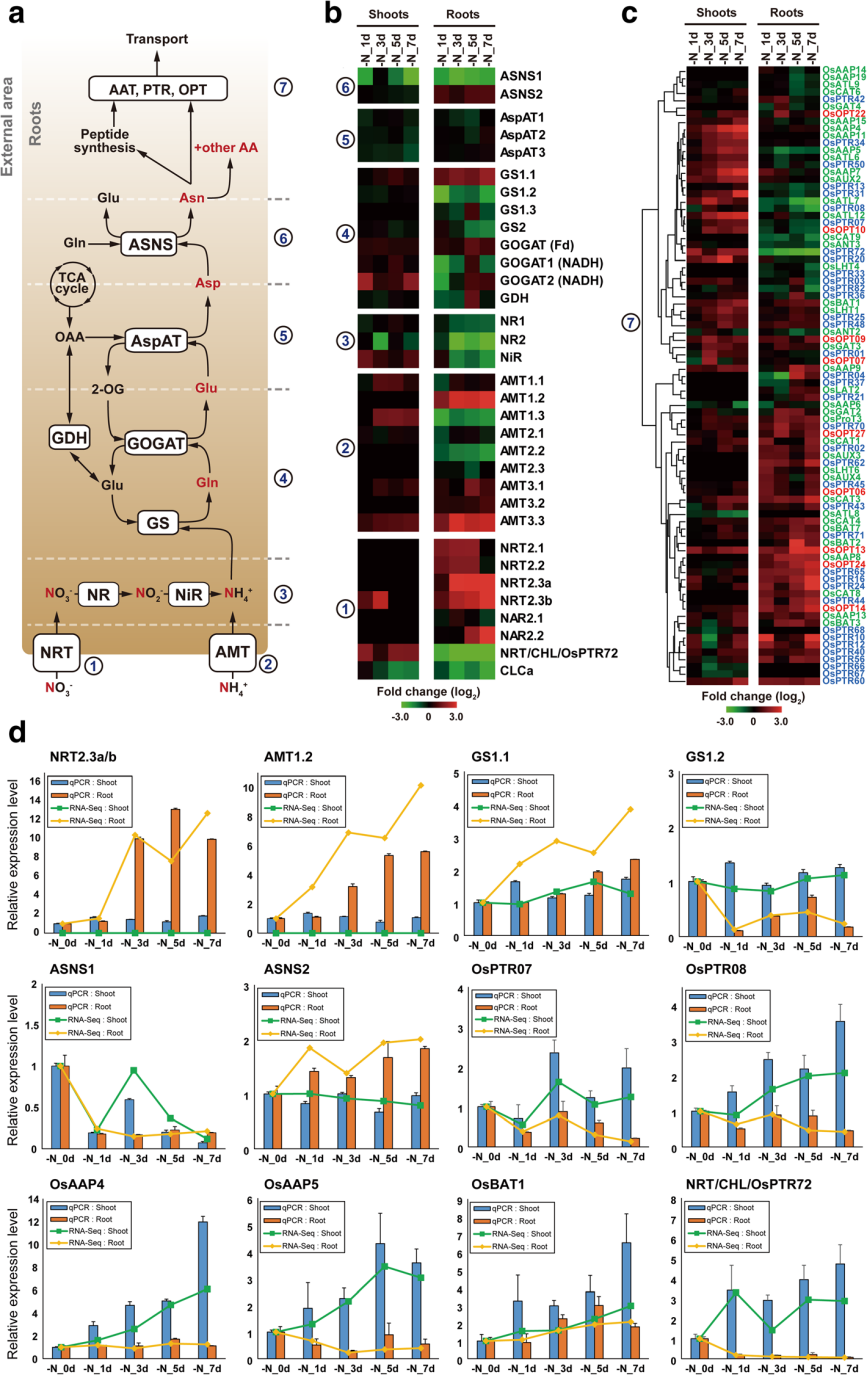
Expression profiles of genes involved in nitrogen (N) source uptake, assimilation, and transport in shoots and roots of N-starved rice. **a** Outline of N uptake, assimilation, and amino acid transport. (1) NRTs (nitrate transporters); (2) AMTs (ammonium transporters); (3) NRs (nitrate reductases) and NiR (nitrite reductase); (4) GSs (glutamine synthetases), GOGATs (glutamine-oxoglutarate aminotransferases), and GDH (glutamate dehydrogenase); (5) AspATs (aspartate aminotransferases); (6) ASNSs (asparagine synthetases); (7) AATs (amino acid transporters), PTRs (peptide transporters), and OPTs (oligopeptide transporters). Glu, glutamate; Gln, glutamine; OAA, oxaloacetate; 2-OG, 2-oxoglutarate; Asp, aspartate; Asn, asparagine. **b** Heatmap visualization of expression profiles of inorganic N source transporters (1–2) and assimilation-involved enzymes (3–6) in N-starved (-N_1d/3d/5d/7d) rice shoots and roots. **c** Expression profiles of 39 amino acid transporters (AATs), 37 peptide transporters (PTRs), and nine oligopeptide transporters (OPTs) in response to N starvation. Genes > 2-fold up- or down-regulated in at least one of four N-starved conditions (-N_1d/3d/5d/7d) in roots and/or shoots are shown. **d** Quantitative RT-PCR analysis of nitrate transporters (NRTs), ammonium transporters (AMTs), glutamine synthetases (GSs), asparagine synthetases (ASNSs), peptide transporters (PTRs), amino acid permeases (AAPs), and oligopeptide transporters (OPTs) in N-starved rice

First, the expression patterns of genes involved in N transport and assimilation were profiled via analysis of RNA-Seq data (Fig. 2a and b), since the transcript abundance of these genes were known to change dynamically in response to N availability [5, 43–47]. Transcript levels of genes encoding nitrate transporters (NRTs) and ammonium transporters (AMTs), varied more dynamically in roots than in shoots in response to N starvation (Fig. 2b). The transcription of *NRT2.1*, *NRT2.2*, *NRT2.3a*, *NRT2.3b*, and *NAR2.2* increased in response to N starvation, whereas that of *NRT/CHL* (Os10g0554200, *AtNRT1.1* homolog) and *CLCa* (Os12g0438600, *AtCLCa* homolog) decreased. The expression of *NRT2.1* and *NRT2.2* continued to increase gradually until 5 days after N starvation, and then reduced to un-induced levels at 7 days after N starvation. The two isoforms of *NRT2.3* were gradually up-regulated until 7 days after N starvation (Fig. 2b and d), suggesting temporal-specific regulation of NRT expression in response to prolonged N starvation. Interestingly, one of the two down-regulated NRTs in both roots and shoots, *CLCa*, is a rice homolog of *AtCLCa*, which specifically localizes to the vacuole membrane and acts as an NO_3_^−^/H^+^ antiporter [48]. Considering that leaf vacuoles function as major nitrate storage pools [49], the down-regulation of *CLCa* might induce the release of vacuole-reserved nitrate to the cytoplasm to maintain nitrate homeostasis in local tissue.

The *AMT1.2* and *AMT3.3* genes showed significant gradual increases in expression over time in N-starved rice roots, while *AMT1.3* and *AMT2.2* showed a gradual reduction in expression levels (Fig. 2b). These results are consistent with previous studies [45, 46]. These reciprocal expression changes in N-starved roots appeared to be part of a pre-programmed response to ammonium deficiency, because many isozymes involved in N assimilation showed similar responses. These results are consistent with previous expression analyses of rice GS, GOGAT, and asparagine synthetase (ASNS) [44, 50–53]. Taken together, these results suggest that genes that are down-regulated in response to N starvation (*AMT1.3*, *GS1.2*, and *ASNS1*) are mainly responsible for primary ammonium assimilation in N-sufficient conditions, whereas genes that are induced by N starvation (*AMT1.2*, *GS1.1*, and *ASNS2*) are active during N-deficient conditions in roots. This is supported by functional characterization studies of GS isozymes and two AMTs in rice [44]. Overall, our profiling results indicate that plants use dynamic reprogramming of gene expression levels to adapt to N-deficient conditions.

### Gene expression profiling of genes involved in amino acid/peptide transport

Effective distribution and localization of amino acids and peptides is necessary for rice growth and development. However, little is known about the genome-wide responses of rice genes encoding amino acid and peptide transporters in N-starved conditions. Here, genes encoding the amino acid transporter (AAT) family and peptide transporter (PTR) family of proteins were profiled in N-starved rice. A total of 191 genes with relevant annotations in Rice Annotation Project Database (RAP-DB; http://rapdb.dna.affrc.go.jp/). RAP-DB were identified; these included 82 out of 85 AAT genes [54], 82 out of 85 PTR genes [55], and 27 additional RAP-DB genes harboring the oligopeptide transporter domains (PF03169.8, E-value >5E-50) (Additional file 2: Table S3). Among these, 68 *AAT*, 68 *PTR*, and 13 *OPT* genes with expression levels > 1.0 FPKM in at least one out of ten N-starved rice RNA-Seq datasets were selected and further analyzed.

Of these genes, the expression of 39 *AAT*, 37 *PTR*, and 9 *OPT* genes was altered by more than 2-fold in at least one out of four N-starved conditions in roots and/or shoots. Hierarchical clustering results showed that AATs/PTRs/OPTs were generally up-regulated both in roots and/or in shoots in response to N starvation (Fig. 2c). Among the genes differentially expressed in 7 day N-starved roots and/or shoots, 31 and 24 genes were up-regulated, whereas only ten and two genes were down-regulated by > 2-fold in roots and shoots, respectively. These results indicated that amino acid/peptide transport systems were activated in roots and shoots in response to N starvation.

Among the amino acid/peptide transporter genes profiled, 64/30 genes were up−/down-regulated in at least one out of eight N-starved rice root and shoot datasets, respectively; 42/24 genes were up−/down-regulated in roots, and 33/9 were up−/down-regulated in shoots. These patterns suggested that most of the AATs/PTRs/OPTs were up-regulated in response to N starvation, and that the dynamic responses of these genes occurred in the roots rather than in the shoots; this was similar to the expression patterns of N metabolism-related genes. Additionally, many of the AATs/PTRs/OPTs showed tissue-specific regulation of expression, especially in roots. While only 15 of the genes exhibited expression changes in both roots and shoots at the same timing in N-starved conditions, 44 and 18 genes exhibited altered gene expression exclusively in roots and shoots, respectively. Tissue-dependent concordant/discordant regulation of some AATs/PTRs/OPTs was also observed. Seven genes showed concordant patterns of up-regulation in both roots and shoots: *OsAAP13* (Os04g0470700), *OsBAT1* (Os01g0607200), *OsBAT3* (Os04g0470700), *OsCAT3* (Os03g0641200), *OsPTR12* (Os01g0872100), *OsOPT09* (Os02g0695800), and *OsOPT13* (Os04g0524500). Conversely, *OsAAP5* (Os01g0878400), *OsATL7* (Os01g0825800), *OsPTR07* (Os01g0871500), and *OsPTR08* (Os01g0871600) were discordantly regulated between two tissues.

Notably, one of the most abundantly expressed PTR genes, *OsPTR07* was down-regulated in roots and up-regulated in shoots. *OsPTR08*, an evolutionary tandem-duplicate of *OsPTR07*, showed similar expression patterns to *OsPTR07* both in N-starved roots and shoots; however, RNA-Seq data showed higher expression of *OsPTR07* compared with *OsPTR08* in RNA-Seq results (Additional file 2: Table S3). According to a previous report, many *OsPTR* genes are thought to be paralogous, and most (~ 67%) are tandemly duplicated; nevertheless, the paralogs appear to be differentially regulated in a tissue-specific manner, suggesting that these tandem duplicates of *OsPTR* genes are undergoing sub-functionalization and neo-functionalization [55]. The different expression levels observed for *OsPTR07* and *OsPTR08* in shoots in response to N starvation may be an example of PTR sub-functionalization. This tissue-dependent concordant/discordant regulation of *OsPTR* genes, including *OsPTR07* and *OsPTR08*, in response to N starvation might allow modulation of local amino acid/peptide transport in rice.

### Profiling N starvation-responsive genes co-regulated by phosphate status

Along with N, inorganic phosphate (Pi) is a key macronutrient that is required for optimal growth and productivity of crop plants. Interactions between the regulatory systems governing these key nutrients are suspected at the physiological and gene expression levels [56–59]. To examine the regulatory effects of limiting N or Pi on homeostasis of the other nutrient at the transcriptome level in rice, we profiled genes responding to both N and Pi starvation using strand-specific RNA-Seq data collected in this study, and non-strand-specific RNA-Seq data obtained from a previous Pi starvation study [60] in which the time course and growth conditions were similar to those used in this study.

**Fig. 3:**
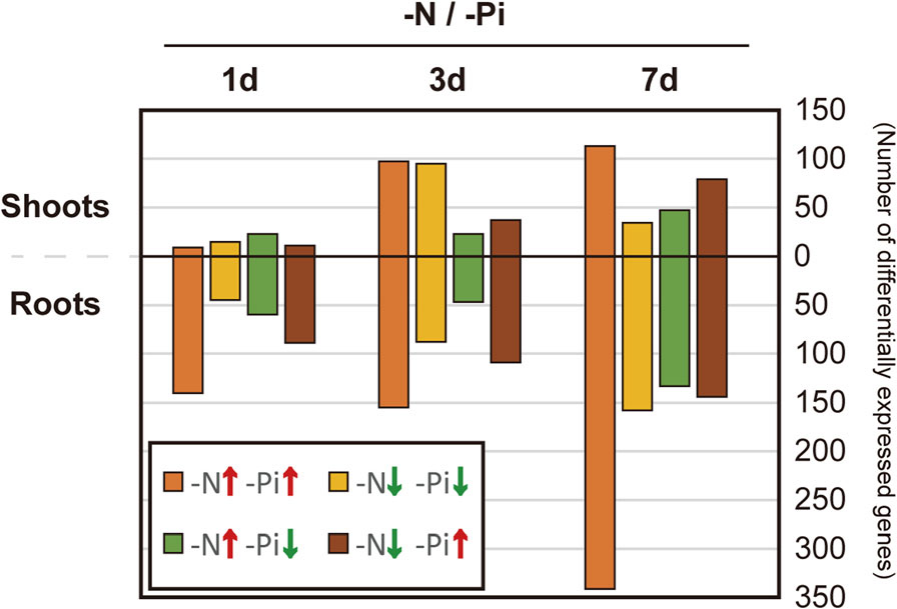
Genes regulated by both nitrogen (N) and phosphate (Pi) starvation in rice. Numbers of genes up−/down-regulated > 2-fold in 1 day, 3 day, and 7 day samples of N-starved (-N) and Pi-starved (-Pi) rice shoots and roots

Expression profiling in 1 day, 3 day, and 7 day N- or Pi-starved rice indicated that some genes involved in the transport or metabolism of each nutrient were partly regulated in response to starvation conditions for the other nutrient. In 7 day N- or Pi-starved rice, 776 genes were up- or down- regulated by both N and Pi starvation (Fig. 3). Several of these genes were involved in N transport and metabolism such as AMTs, amino acid/peptide transporters, and N assimilating enzymes, and were also regulated by Pi starvation in roots (Table 1). Furthermore, Pi starvation-responsive genes like *SPX2* and *IPS1* were regulated in the roots of N-starved rice. Both *AMT1.2* and *AMT1.3* were down-regulated in Pi-starved roots, whereas only *AMT1.2* was up-regulated in N-starved roots. *IPS1*, which is induced by Pi starvation in roots and in shoots of rice [60], exhibited discordant expression patterns between shoot and root in response to N starvation (Table 1). Collectively, these expression patterns suggested that, although the regulatory effects of N or Pi starvation on the metabolic pathways of the other nutrient appeared to be locally limited or partial, deficiency of N or Pi could regulate transcription of genes involved in their own and the other nutrient’s metabolic pathways.

**Table 1:** List of genes regulated by both nitrogen and phosphate starvation in rice roots and shoots

Gene ID	Description	Fold Change (log_2_)	
Root_1d		-N_1d / -N_0d	-Pi_1d / +Pi_1h
Os03g0150800	PT2	−1.68	1.67
Root_3d		-N_3d / -N_0d	-Pi_3d / +Pi_1h
Os01g0748950	OsPTR04	−3.58	−2.22
Os03g0641200	OsCAT3	1.44	1.52
Os06g0569500	Similar to Ent-kaurene oxidase 1	1.60	2.18
Os10g0172100	Similar to Cytochrome P450 family protein, expressed	3.23	5.26
Root_7d		-N_7d / -N_0d	-Pi_7d / +Pi_1h
Os02g0620500	AMT1.2	3.34	−14.40
Os02g0620600	AMT1.3	−1.92	−2.37
Os02g0550800	AMT3.3	2.63	2.42
Os01g0882800	OsAAP8	1.75	1.56
Os01g0825800	OsATL7	−3.52	−1.81
Os01g0871500	OsPTR07	−1.23	−1.38
Os01g0871600	OsPTR08	−2.91	−5.09
Os01g0902700	OsPTR17	3.92	3.53
Os03g0223400	GS1.2	−2.11	−1.78
Os01g0682001	GOGAT1(NADH)	−1.29	−2.42
Os03g0291500	ASNS1	−2.24	−1.38
Os02g0202200	SPX2	1.39	4.11
Os03g0146800	IPS1	−4.40	12.09
Os01g0719300	Similar to Sulfate transporter 3.1	1.79	2.01
Os03g0195450	Similar to sulfate/bicarbonate/oxalate exchanger and transporter sat-1	−1.65	−1.15
Os02g0776400	Similar to nuclear transcription factor Y subunit A-3	1.19	1.37
Os07g0608200	NF-YA6	1.30	1.58
Os01g0187600	OsCKX1	1.41	1.03
Os01g0940000	OsCKX4	1.16	1.86
Shoot_1d		-N_1d / -N_0d	-Pi_1d / +Pi_1h
Os04g0524500	OsOPT09	1.43	−1.39
Os03g0291500	ASNS1	−2.14	−2.07
Shoot_3d		-N_3d / -N_0d	-Pi_3d / +Pi_1h
Os02g0809800	PHO1;2	1.94	1.11
Os02g0578400	PsbQ family protein	−1.37	−2.36
Os08g0191900	Pentatricopeptide repeat domain containing protein	−1.30	−2.33
Shoot_7d		-N_7d / -N_0d	-Pi_7d / +Pi_1h
Os01g0547600	NRT2.4	1.88	1.39
Os02g0102200	OsAAP9	1.17	1.09
Os06g0228500	OsATL12	2.30	1.89
Os03g0406100	SPX5	−2.77	3.61
Os04g0186400	PT4	1.40	2.60
Os06g0493600	PHO1;3	−1.38	1.61
Os03g0146800	IPS1	1.28	4.76
Os01g0194600	Thioredoxin fold domain containing protein (OsGrx_A2)	1.27	1.25
Os05g0198200	Thioredoxin fold domain containing protein (OsGrx_C15)	1.02	1.48
Os10g0482900	Thioredoxin fold domain containing protein	2.11	1.07
Os03g0288000	Similar to Metallothionein	3.21	2.46
Os05g0202800	Similar to Metallothionein-like protein 3B	2.95	1.31

The set of genes exhibiting responses to both N and Pi starvation was examined using GO analysis. GO results showed that genes up-regulated in 7 day N- and Pi-starved rice shoots were enriched for cellular homeostasis (GO:0019725) (Additional file 1: Figure. S2). Five of the six cellular homeostasis genes encoding thioredoxin fold domain-containing proteins (Os01g0194600, Os05g0198200, Os10g0482900) and two metallothioneins (Os03g0288000, Os050202800), were previously shown to be involved in antioxidant activity [61–63]. Oxidative stress is induced under nutrient-deficient conditions [64] and can cause deleterious damage to important metabolic enzymes such as GS [62]. Increased expression of antioxidant genes in N- or Pi-starved conditions might provide protection from damage by scavenging harmful oxidants two cytokinin oxidase/dehydrogenases (*OsCKX1*, *OsCKX4*) involved in cytokinin degradation are up-regulated in 7 day N- and Pi-starved rice roots. The *in planta* status of cytokinin can affect the expression of genes involved in N and Pi pathways [65, 66]. N and Pi starvation may also induce cytokinin degradation, leading to modulation of root-to-shoot growth ratio and lateral root development [67, 68]. Induction of *CKX* genes in rice roots might be involved in regulating cytokinin status in response to N and Pi starvation.

### Identification and profiling of putative lncRNAs under abiotic stress conditions, and characterization of the 3′-end of transcripts using 2P-Seq

**Fig. 4:**
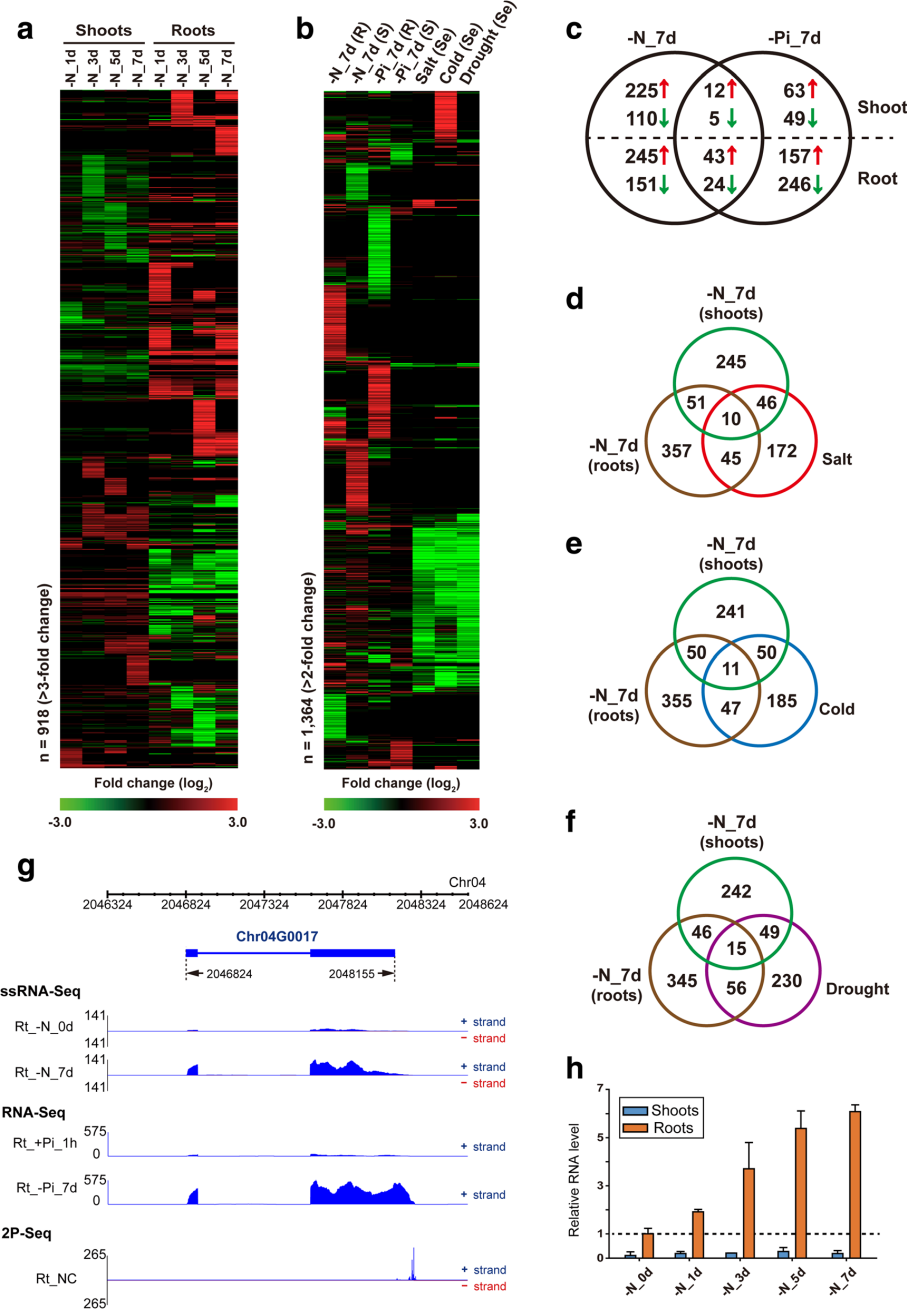
Expression profiling of putative lncRNAs under nitrogen (N) starvation and other stress conditions in rice. **a** Heatmap representation of low-coding potential novel transcripts differentially expressed in N-starved (-N_1d/3d/5d/7d) rice shoots and roots. **b** Heatmap representation of low-coding potential novel transcripts responding to various stress conditions. R, roots; S, shoots; Se, seedling. **c** Venn diagram of low-coding potential novel transcripts responding 7 day of N starvation (-N_7d) and phosphate starvation (-Pi_7d). Red arrow, > 2-fold up-regulation; green arrow, > 2-fold down-regulation. **d, e, f** Venn diagrams of putative lncRNAs responding to 7 days of N starvation (-N_7d), 7 days of phosphate starvation (-Pi_7d), and salt (**d**), cold (**e**), or drought (**f**) stress. **g** Read distribution of a N and phosphate (Pi) starvation-responsive putative lncRNA, Chr04G0017 in N-starved (-N) and Pi-starved (-Pi) rice. **h** Quantitative RT-PCR of Chr04G0017 in N-starved (-N) rice shoots and roots. Both shoots and roots are normalized relative to *eEF-1α*. N.D., not detected by qRT-PCR

After RABT assembly and a stepwise filtering, we identified a total of 2588 putative lncRNA-encoding loci (Fig. 1b, Additional file 2: Table S4). During the CPC value calculation step, we noticed that the rice protein sequences in the SwissProt non-redundant (NR) database included a number of short peptide sequences that were non-conserved and were not experimentally validated. This removed ~ 20% of the assembled transcripts, and we thus re-evaluated the CPC values of the filtered-out transcripts with SwissProt NR database that *Oryza* protein sequences were excluded, which resulted in the inclusion of 594 additional loci in our lncRNA dataset. To distinguish transcripts filtered by the different CPC evaluation processes, the re-evaluated lncRNAs were denoted as relatively high-coding (RHC) potential and relatively low-coding (RLC) potential lncRNAs. Among the lncRNA transcripts, 1255 and 1333 were expressed from intergenic regions and *cis*-NATs, respectively (Fig. 1b), and mostly comprised of single-exon transcripts (~ 74%). A large number of loci (918) were up- or down-regulated in response to N starvation in rice shoots and roots (Fig. 4a). Similar to the aforementioned profiling results for N metabolism and transport genes, many putative lncRNA loci showed more significant expression changes in N-starved roots than shoots (Fig. 4a), indicating that their responses to N starvation were tissue-dependent.

RNA-Seq data derived from rice samples subjected to Pi starvation and abiotic stress [60, 69] were analyzed to investigate stress-related characteristics of putative lncRNAs. Many of the putative lncRNAs that were unresponsive to N starvation were responsive to other stress conditions (Fig. 4b). A small number of putative lncRNAs were concordantly regulated in response to N and Pi starvation in 7 day rice samples (Fig. 4c). Analysis of N starvation and salt-, cold-, and drought-responsive transcription also showed a similar tendency (Fig. 4d–f). Some lncRNAs were specifically induced by cold stress, but the majority of these were down-regulated by salt and drought stress conditions (Fig. 4b). One of these cold stress-responsive putative lncRNAs, Chr03G0008, was expressed only in cold-treated seedlings (Additional file 1: Figure S3A). Chr03G0008 also showed N starvation- responsive induced expression patterns in N-starved roots, suggesting that Chr03G0008 responded to multiple stress conditions.

Expression of a well-known lncRNA, *IPS1*, changed in response to both N and Pi starvation (Table 1), suggesting that *IPS1* might play a bridging role between the pathways regulating the two macronutrients. Similar putative lncRNAs that were co-regulated by both N and Pi deficiency were identified in our dataset. Expression of one such RHC-class lncRNA, Chr04G0017, was induced following both N and Pi starvation, especially in rice roots (Fig. 4g and h). Chr04G0017 comprised two exons with one distinct 2P-Seq signal at the 3′-end region, indicating strong polyadenylation. In the dataset analyzed by Secco et al., Chr04G0017 was ~ 10-fold induced after 21 days of Pi starvation [60]. Polysomal fractionation analysis by Secco et al. suggested that Chr04G0017 [60] was actively translated, which was consistent with the classification of Chr04G0017 as a RHC-class lncRNA in our filtering pipeline. Circumstantial and experimental evidence suggests that Chr04G0017 acts via its encoded peptide (116 a.a., 13.64 kDa) in nutrient-starved conditions. In a final example, expression of alternatively polyadenylated Chr07G0166 was induced after 7 days of both N and Pi starvation (Additional file 1: Figure S3B and S4E).

**Fig. 5:**
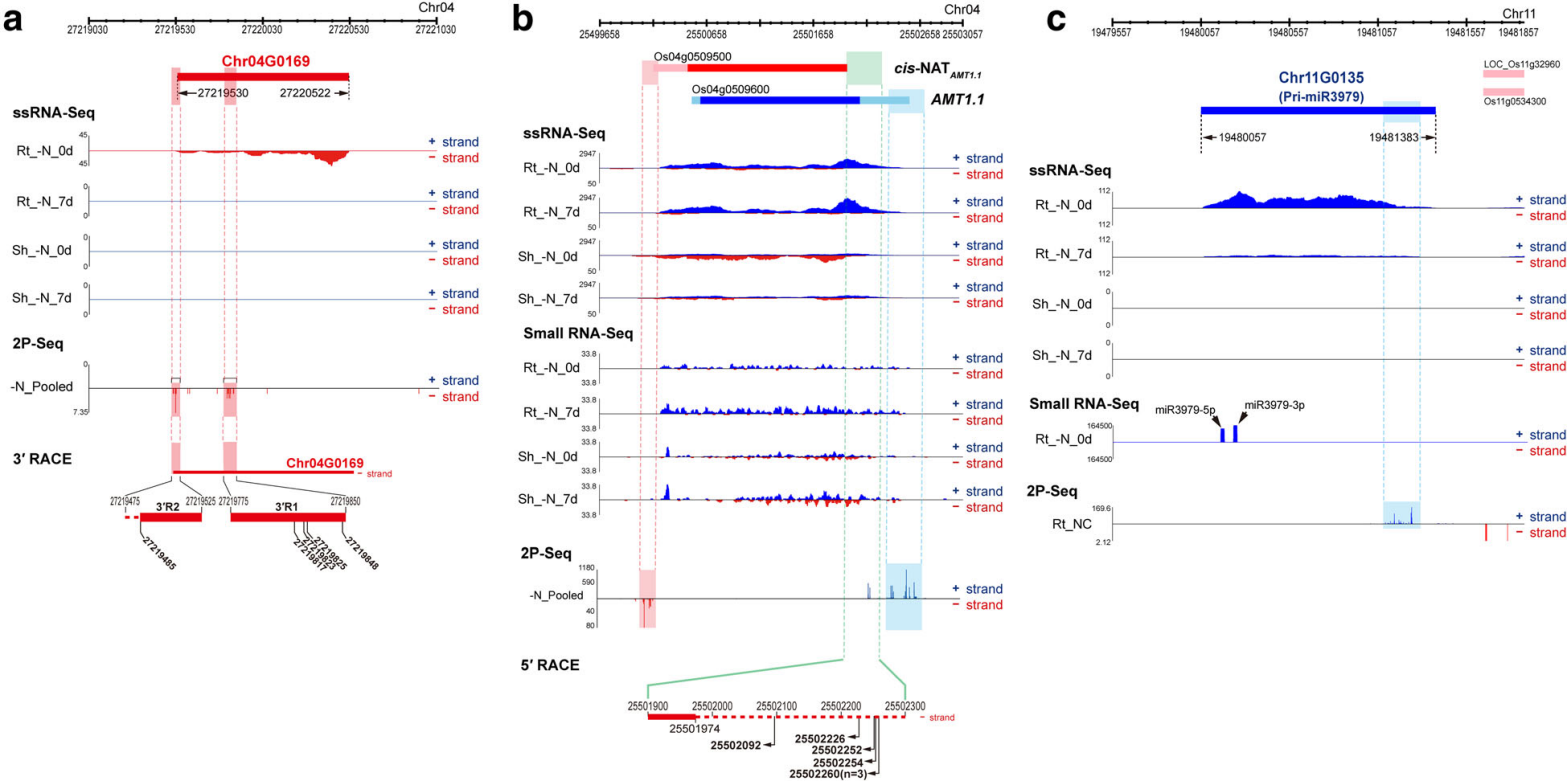
Transcript models and their 3′ ends of annotated genes, putative lncRNAs and primary microRNAs from read distribution of transcriptome data. **a** Read distribution and 3′ RACE of N starvation-responsive putative lncRNA, Chr04G0169, in N-starved (-N) rice. **b** Read distribution for the genomic region harboring *AMT1.1* and *cis-*NAT_*AMT1.1*_ in N-starved (-N) rice and 5′ RACE of *cis-*NAT_*AMT1.1*_. Rt, root; Sh, shoot. **c** Read distribution of N starvation-responsive primary microRNA, pri-miR3979 (Chr11G0135) in N-starved (-N) rice

Although previous studies examined the expression of annotated genes and identified novel transcripts in rice, no studies have as yet defined or characterized the 3′-end of transcripts. Here, we used 2P-Seq analysis redefine models of various transcripts, including annotated genes, putative lncRNA loci, and primary miRNA (pri-miRNA) transcripts, with the aim to provide accurate models for future research. The 2P-Seq dataset examined sequences upstream of the poly A tail at the 3′ ends of transcripts. The results of 2P-Seq analysis revealed 647 (25%) putative lncRNAs with significant 2P-Seq signals on transcript models near the 3′ ends. Detailed scrutiny of N starvation-responsive putative lncRNAs revealed that a small number of these lncRNAs exhibited 3′ end-related characteristics, some of these were alternatively polyadenylated. One putative lncRNA, Chr04G0169, which was expressed exclusively in roots, showed two distinct groups of 2P-Seq signals at the 3′-end region: these signals were further validated by 3′ RACE (Fig. 5a). Another putative lncRNA, Chr07G0166, was present as three isoforms with different 3′ ends. Distances between 2P-Seq signals were ~ 1 kb, with similar distances between the polyadenylation sites of the short and long isoforms (Additional file 1: Figure S3B).

Integrated analysis of strand-specific RNA-Seq and 2P-Seq datasets also allowed the revision of transcript models, including those of previously annotated genes and pri-miRNAs. The expression of *AMT1.1* and *cis*-NAT_*AMT1.1*_ was detected by both strand-specific RNA-Seq and 2P-Seq (Fig. 5b). Read distribution and 5′ RACE identified 5′ extended regions of *cis*-NAT_*AMT1.1*_ (Fig. 5b). The polyadenylation sites of *AMT1.2* and *cis*-NAT_*AMT1.2*_ transcripts were also detected by 2P-Seq, which showed longer 3′-ends of these transcripts than the IRGSP gene models (Additional file 1: Figure. S7A). In the case of pri-miR3979, two significant 3′ polyadenylation sites were observed for the single-exon transcript model of pri-miR3979 (Fig. 5c).

### Analysis of N starvation-responsive miRNAs and their targets

**Fig. 6:**
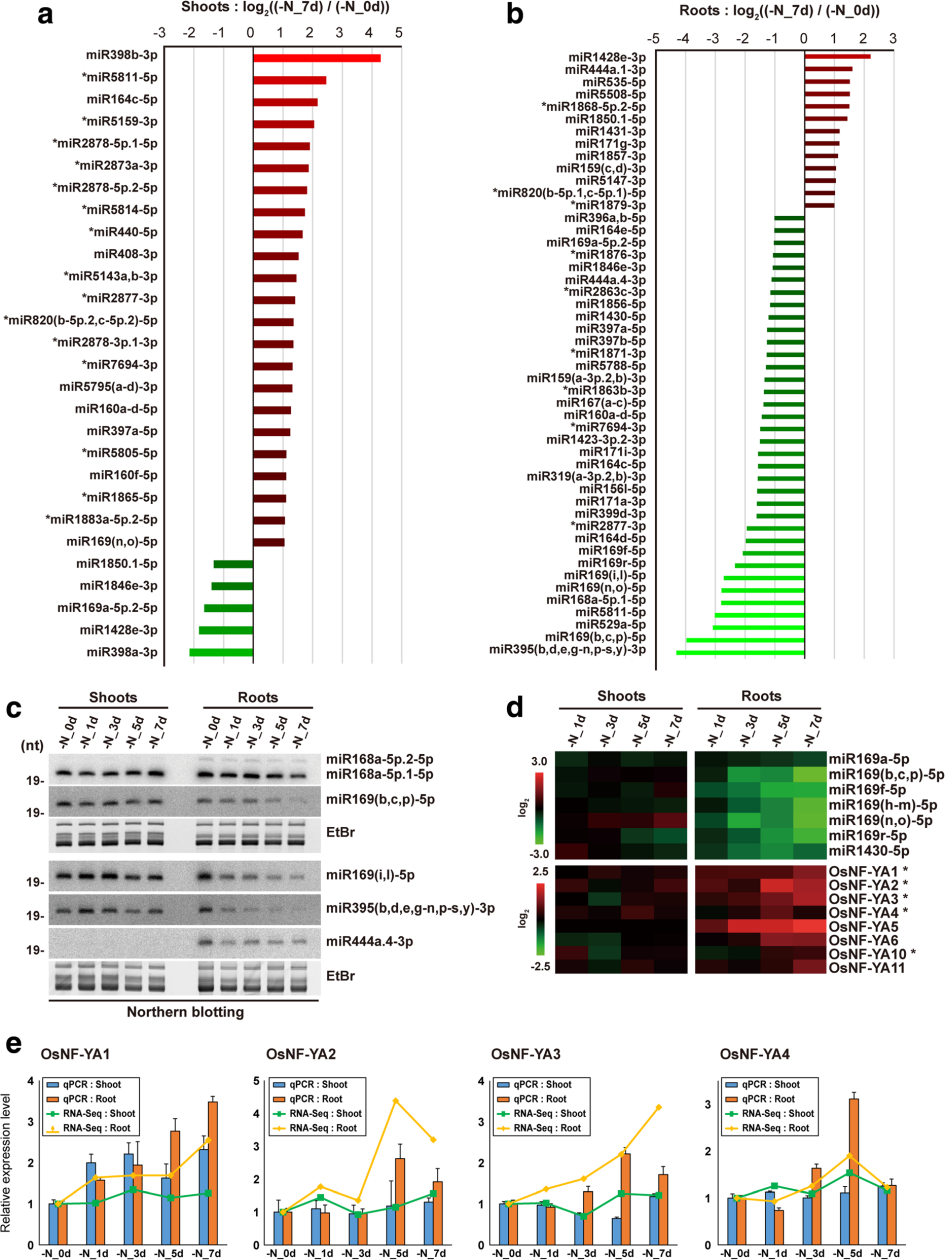
Expression profiles of nitrogen (N) starvation-responsive microRNAs in rice shoots and roots. **a** and **b** Differential expression of rice microRNAs (miRNAs) between 0 day (-N_0d) and 7 day (-N_7d) of N starvation in rice shoots (**a**) and roots (**b**). Fold changes of miRNAs with > 2-fold up- or down-regulation are represented using a log_2_ scale. **c** Northern blot analysis of microRNAs in N-starved rice shoots and roots. **d** Heatmap showing the expression level of osa-miR169 family and their *OsNF-YA* target genes in N-starved rice shoots and roots. **e** Relative expression level of four *OsNF-YA* genes measured by RNA-Seq and qRT-PCR in N-starved (-N) rice shoots and roots

The most substantial changes in miRNA expression were observed in 7 day N-starved samples (Fig. 6a and b). Shoots and roots of 7 day N-starved rice exhibited differential miRNA expression patterns. The majority of differentially expressed miRNAs were up-regulated in shoots of 7 day N-starved rice (Fig. 6a), and only five miRNAs were down-regulated. Almost three-fourths of differentially expressed miRNAs were down-regulated in roots of 7 day N-starved rice (Fig. 6b). Of these, members of the osa-miR169 family were significantly down-regulated in response to N starvation (Fig. 6c and d). Most of the osa-miR169 family exhibited increasing down-regulation over time in N-starved roots (Fig. 6d). Target prediction for osa-miR169 indicated that 8 of 11 rice genes encoding the nuclear transcription factor Y subunit alpha (NF-YA) were predicted to be targeted (Additional file 1: Figure S5A), and their expression levels were up-regulated in response to N starvation in rice roots (Fig. 6d and e). To confirm osa-miR169-mediated post-transcriptional regulation of NF-YAs, 5′ RACE was performed against all predicted NF-YA target genes. The 5′ RACE results showed that 7 of 8 NF-YAs were regulated by miR169-mediated cleavage (Additional file 1: Figure. S5C–I). These results suggested that up-regulation of NF-YAs in N-starved rice roots was the result of osa-miR169 de-repression in N starvation conditions.

We also observed the down-regulation of the osa-miR395 family and osa-miR399d-3p (Fig. 6b and c), which are responsible for modulating sulfur and Pi homeostasis, respectively, in response to changes in the sulfur/Pi supply status. Down-regulation of these miRNAs was consistent with previous reports from Arabidopsis and maize [35, 36, 38], and suggested that N starvation impacted the homeostasis of other nutrients regulated by nutrient-responsive miRNAs. Finally, root-specific expression of osa-miR444a.4-3p, one of the most abundant root miRNAs in our dataset, was also down-regulated in response to N starvation (Fig. 6b and c).

**Fig. 7:**
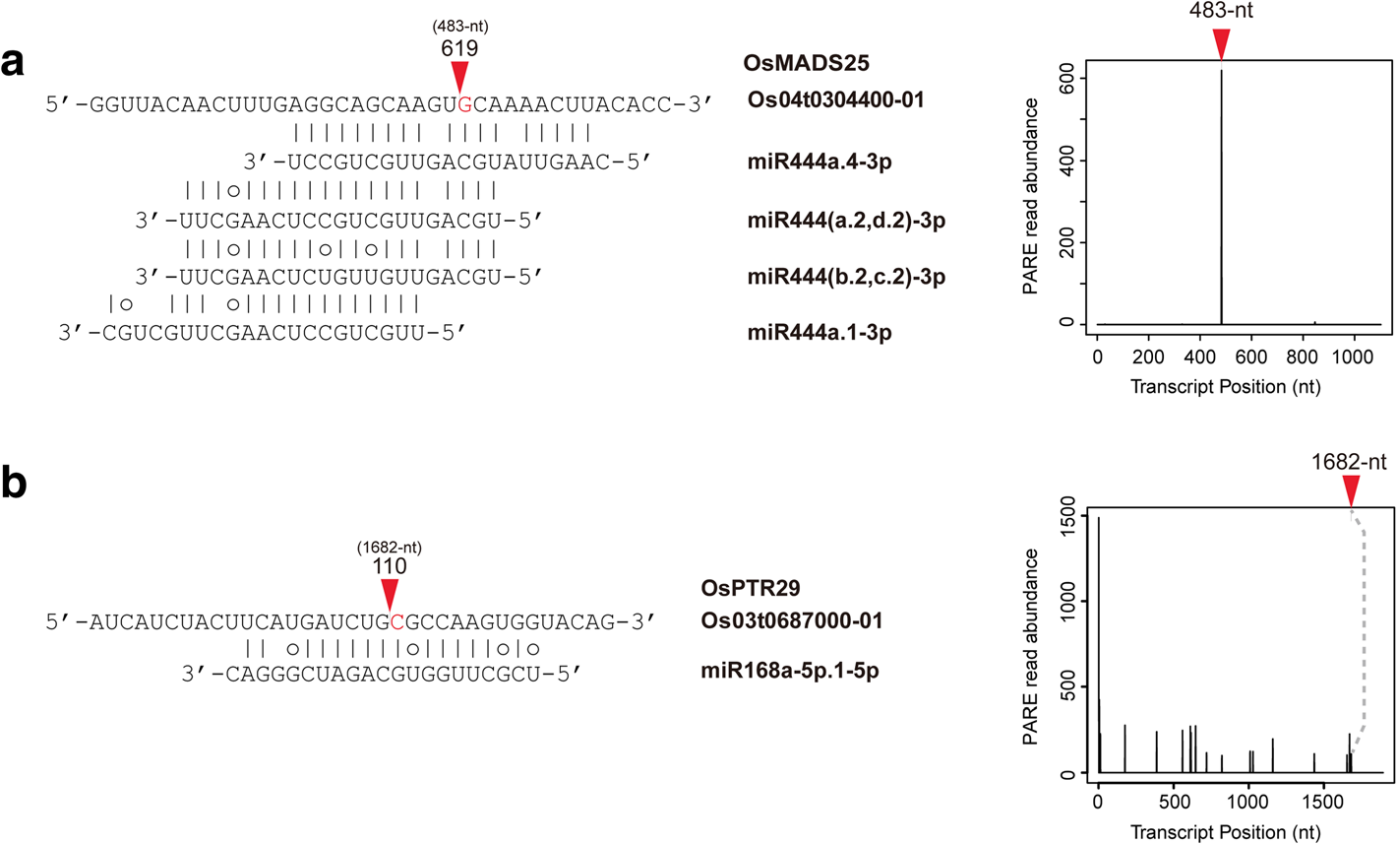
Degradome data analysis results of **a** *OsMADS25* and **b** *OsPTR29*. Red arrow indicates predicted microRNA-cleavage site. Bars in boxed graphs indicate raw Degradome sequencing read numbers aligned to the target transcripts

To examine the effect of changes in miRNA expression on target mRNAs, Degradome sequencing data from 7 day N-starved rice roots were analyzed using the CleaveLand pipeline. In total, 91 rice transcripts were identified as targets of 40 miRNA sequences (Additional file 2: Table S7). Several differentially expressed miRNAs were found to regulate their own target transcripts in 7 day N-starved rice roots (Table 2). One target gene identified by Degradome data analysis, *OsMADS25*, was shown to be targeted by osa-miR444a.4-3p (Fig. 7a). Osa-miR444 family sequences were derived from the NATs of four *ANR1*-like MADS-box genes (*OsMADS23*, *OsMADS27a*, *OsMADS27b*, *OsMADS57*) and were shown to target all four MADS-box genes [40, 70, 71]. However, osa-miR444a.4-3p-mediated regulation of *OsMADS25* was not confirmed in a previous Degradome analysis of rice [72]. In this study, Degradome analysis showed that only osa-miR444a.4-3p cleaved *OsMADS25* (Fig. 7a), and osa-miR444a.4-3p only seemed to target *OsMADS25*. Expression levels of osa-miR444a.4-3p and *OsMADS25* were negatively correlated in N-starved rice, suggesting that N starvation-mediated down-regulation of osa-miR444a.4-3p affected up-regulation of *OsMADS25*. In addition, osa-miR168a-5p.1-5p was involved in mediating the *OsPTR29* cleavage pattern (Fig. 7b). *OsPTR29*, also known as *OsNPF2.4*, is a pH-dependent, low-affinity NRT that plays a role in nitrate acquisition and long-distance nitrate transport [6].

**Table 2:** Degradome sequencing results from 7 day N-starved rice roots

	Roots sRNA-Seq reads (RP40M)									
miRNA	-N_0d	-N_1d	-N_7d	Target transcript ID (Category)	PenaltyScore (≤5.0)	Description	Cleavage site (nt)	Cleaved reads (RPM)	Reads on cleavage site (%)	*p-Value*
Osa-miR156(a-j)-5p	908,961.7	1,151,055	968,459.5	Os02t0174100–01 (III)	1	SPL4	2231	0.41	2.28	1.1.E-02
				Os06t0663500–00 (0)	1	SPL11	759	15.52	34.32	1.4.E-03
				Os06t0703500–03 (II)	1	SPL12	1095	3.55	26.71	3.1.E-03
Osa-miR156k-5p	1099.9	2027.7	1218.8	Os01t0922600–01 (0)	0	SPL2	630	85.88	76.18	1.4.E-03
				Os02t0139400–01 (II)	1	SPL3	1879	9.24	16.69	1.5.E-02
				Os09t0507100–00 (0)	0	SPL18	1044	63.53	59.46	3.4.E-03
Osa-miR159(a-3p.2, b)-3p ^a^	21,468.9	12,155.8	8436	Os06t0605600–01 (0)	3.5	GAMYBL1	414	18.57	68.28	5.6.E-04
				Os11t0569600–01 (0)	5	Receptor kinase-like protein, leucine-rich repeat-containing	656	10.38	61.79	4.2.E-03
Osa-miR159(c, d)-3p ^a^	314.6	469.5	652.3	Os03t0785800–01 (IV)	3.5	OsPCF6	1193	0.02	0.36	3.4.E-03
Osa-miR159f-3p	8930.5	6188	7552.9	Os01t0812000–03 (0)	2.5	OsGAMYB	1268	15.23	32.03	2.8.E-04
Osa-miR160(a-d)-5p ^a^	7484.8	2521.3	2763.7	Os02t0628600–01 (0)	1	ARF8	1411	515.20	84.3	2.8.E-04
Osa-miR160e-5p	7351.4	4602.3	5552.2	Os04t0519700–01 (0)	1	ARF10	1496	110.12	90.26	2.8.E-04
				Os06t0685700–01 (0)	1	ARF18	2006	105.70	56.83	8.4.E-04
				Os10t0479900–01 (0)	1	ARF22	1649	105.68	82.54	5.6.E-04
Osa-miR164(a, b, f)-5p	24,702	21,252.1	15,538.9	Os02t0579000–01 (0)	2	NAC1	752	11.70	20.54	1.7.E-03
				Os04t0460600–01 (II)	2	NAC2	923	10.74	20.03	4.1.E-03
				Os06t0675600–01 (III)	2	NAC11	965	0.20	100	3.1.E-03
Osa-miR164d-5p ^a^	41,779.7	16,096	10,610.4	Os04t0460600–02 (II)	2	NAC2	801	11.63	21.05	5.1.E-03
				Os12t0610600–01 (0)	1	NAC60	922	65.67	54.15	8.4.E-04
Osa-miR168(a-5p.1)-5p ^a^	183,169.3	64,155.2	26,233.1	Os02t0672200–01 (0)	5	AGO1a	616	17.94	17.62	4.7.E-03
				Os03t0687000–01 (III)	5	NPF2.4/OsPTR29 ^c^	1682	2.50	2.14	2.2.E-01
				Os04t0566500–02 (0)	5	AGO1b	708	3.71	9.88	3.4.E-03
Osa-miR169f-5p ^a^	3214.3	1219.4	761	Os03t0411100–02 (II)	3	NF-YA2	1252	3.94	18.19	1.1.E-02
Osa-miR169(n, o)-5p ^a^	2129	503.9	307.1	Os03t0696300–02 (II)	3	NF-YA4	469	5.24	35.11	8.2.E-03
				Os12t0618600–01 (0)	1.5	NF-YA10	1188	45.75	58.28	1.4.E-03
Osa-miR169r-5p ^a^	2816.4	1375.9	556.9	Os03t0174900–01 (III)	4	NF-YA1	1004	5.35	11.08	4.6.E-03
				Os03t0647600–01 (III)	3.5	NF-YA3	1729	4.12	10.12	6.1.E-03
Osa-miR171a-3p ^a^	10,020	3781.9	3301.6	Os02t0663100–01 (0)	1	GRAS transcription factor	1531	31.30	34.69	1.7.E-03
				Os06t0105350–00 (II)	0.5	Similar to Scarecrow-like 6	476	10.95	26.16	1.0.E-03
Osa-miR171i-3p ^a^	1172.8	705.1	396.7	Os04t0555000–01 (II)	1	GRAS transcription factor	1332	22.01	26.92	3.1.E-03
Osa-miR172(a, d)-3p ^a^	520.8	227	568.4	Os05t0121600–01 (III)	2	AP2/EREBP family transcription factor	1423	5.10	1.95	3.1.E-03
Osa-miR319(a-3p.2,b)-3p	6489	3137	2180.1	Os03t0785800–01 (0)	1.5	OsPCF6	1192	6.28	99.64	2.8.E-04
				Os07t0152000–00 (0)	1.5	TCP21	1265	24.95	45.95	5.6.E-04
Osa-miR393(a, b)-5p	20,525.3	30,023.2	37,524.4	Os04t0395600–02 (0)	1	Auxin signaling F-box 2	1789	8.38	62.37	1.1.E-03
				Os05t0150500–00 (0)	1	OsTIR1	1566	50.80	40.65	2.8.E-04
Osa-miR396(a, b)-5p ^a^	1622.8	866.8	803	Os12t0484900–01 (0)	4	OsGRF7	743	15.18	86.51	8.4.E-04
Osa-miR444(b.1,c.1)-3p	110,596.5	92,299	126,685.4	Os04t0461300–01 (II)	0	MADS27b	1204	4.85	21.96	4.1.E-03
Osa-miR444(b.2,c.2)-3p	152,234.5	199,621.8	95,687.8	Os02t0579600–00 (0)	0	MADS27a	575	19.64	67.16	2.8.E-04
				Os04t0461300–01 (0)	0	MADS27b	1210	10.77	48.76	1.1.E-03
Osa-miR444a.4-3p ^a^	242,647.4	197,955.4	91,760.7	Os04t0304400–01 (0)	5	MADS25 ^c^	483	14.09	98.72	3.3.E-02
Osa-miR1425-5p ^b^	120,499.8	100,180.7	90,360.7	Os10t0167600–01 (II)	5	Similar to CPD photolyase	870	5.05	30.41	1.8.E-02
				Os10t0495200–02 (III)	4.5	RF1 (PPR domain containing protein)	1307	3.73	100	1.1.E-02
				Os10t0495400–01 (III)	4.5	PPR domain containing protein	118	4.14	29.45	1.2.E-02
				Os10t0497300–01 (0)	3.5	PPR domain containing protein	1319	5.87	55.36	2.8.E-04
Osa-miR3979-3p ^b^	131,109.3	216,357.6	110,742.2	Os07t0513200–01 (0)	5	PPR domain containing protein	768	4.26	95.89	2.8.E-02

^a^N-responsive miRNAs detected in small RNA-Seq

^b^miRNAs whose primary transcripts are N-responsive in RNA-Seq

^c^Previously non-annotated target genes

Some of the miRNAs that did not exhibit a significant change in the small RNA-Seq dataset were shown to regulate target genes through cleavage mechanisms (Additional file 1: Figure S6). The osa-miR1425-5p, which targeted genes encoding pentatricopeptide repeat (PPR) proteins (Additional file 1: Figure. S6C), was abundantly expressed in roots. Both osa-miR1425-5p and its primary transcript were slightly down-regulated in 7 day N-starved roots (Table 2). PPR is an organellar RNA-binding protein that participates in RNA editing in chloroplasts and mitochondria [73, 74]. Previously, functional analysis of mitochondrial PPRs in *Arabidopsis* has demonstrated that these proteins are involved in regulating root growth, energy metabolism, and abiotic stress responses [75–78]. These results suggest that osa-miR1425-5p has a potential role in modulating root architecture and energy metabolism in roots of rice plants.

### Expression of *cis*-NATs and small RNAs at the genomic regions of *AMT1* genes

Previous reports suggested that some *cis*-NATs could regulate target genes on the antisense genomic region corresponding to their protein-coding genes [79–81]. We examined whether genes involved in N metabolism and transport transcribed potential regulatory *cis*-NATs. Eight of nine AMT genes had IRGSP-annotated *cis*-NATs. In our strand-specific RNA-Seq dataset, most of these *cis*-NAT_AMT_s were expressed at very low levels (< 0.1 FPKM) or were not detected. However, two *cis*-NAT_AMT_s, *cis*-NAT_*AMT1.1*_ (Os04g0509500) and *cis*-NAT_*AMT1.2*_ (Os02g0620533), showed relatively high expression (> 1 FPKM), and their 3′-end poly A tails were supported by 2P-Seq (Fig. 5a and Additional file 1: Figure. S7A). These two polyadenylated *cis*-NAT_*AMT1*_ transcripts and the corresponding *AMT1* genes showed different expression patterns: *cis*-NAT_*AMT1.2*_ and *AMT1.2* were exclusively expressed in rice roots, and both showed increased expression levels in response to N starvation, whereas *AMT1.1* and *cis*-NAT_*AMT1.1*_ showed tissue-dependent discordant expression patterns. *AMT1.1* was expressed more abundantly in roots, whereas *cis*-NAT_*AMT1.1*_ was rarely expressed in roots and was expressed more abundantly in shoots (Fig. 5a).

During observation of RNA-Seq read distribution on the genomic region of *AMT1.1* and *AMT1.2*, we observed that a few small RNA reads aligned to the genomic region of two *AMT1* family genes and had expression patterns that generally correlated with the expression patterns of AMT genes and/or their *cis*-NATs described above (Fig. 5b and Additional file 1: Figure. S7A). First, we assumed that these small RNAs came from double-stranded RNAs (dsRNAs) consisting of *cis*-NAT pairs, since the genomic region of these genes overlapped extensively. However, expression patterns of *AMT1* genes, their *cis*-NATs, and small RNAs suggest that these small RNAs are derived from single-stranded RNA of *AMT1*s and their *cis* -NATs. In the case of small RNAs in the *AMT1.1* genomic region, expression levels of (+)-strand small RNAs correlated with expression levels of *AMT1.1* rather than those of *cis*-NAT_*AMT1.1*_ (Fig. 5a). Similarly, (+)-strand small RNAs sense to *AMT1.2* were induced in concordance with *AMT1.2* transcript levels in N-starved rice roots (Additional file 1: Figure. S7A). However, although expression patterns of *cis*-NAT_*AMT1.1*_ and corresponding (−)-strand small RNAs were concordant in a tissue-dependent manner, *cis*-NAT_*AMT1.1*_ and the corresponding (−)-strand small RNAs were not concordantly regulated in response to N starvation. Moreover, in silico RNA structure analysis of the homologous region of *AMT1.1* and *AMT1.2* sequences showed potent stem-loop structures that were sufficient to produce small RNAs (Additional file 1: Figure. S7B and S7C). Together, these results suggested that the small RNAs aligned to the genomic regions of *AMT1.1* and *AMT1.2* were derived from sense transcripts.

## Discussion

In this study, we profiled and characterized multiple aspects of the rice transcriptome in N-starved rice plants in a spatio-temporal manner, via four different transcriptomic approaches. Expression patterns of genes involved in N metabolism and transport were characterized, and a set of putative lncRNA loci was identified. In addition, entities and characteristics of 3′-ends of rice transcripts were identified and analyzed by 2P-Seq. We also profiled annotated rice genes and putative lncRNAs in Pi starvation-treated rice samples to gain insights into the relationships between gene regulatory pathways involved in the metabolism of N and other nutrients. Comparative analysis of RNA-Seq datasets from N- and Pi-starved rice identified co-regulated genes and putative lncRNAs, and one of those lncRNAs (Chr04G0017) was identified to be regulated by both N and Pi starvation in a root-specific manner. Additionally, analysis of putative lncRNAs in Pi-starved samples as well as salt-, cold-, and drought-stressed rice samples suggested that a set of putative lncRNAs was regulated in a stress-specific manner. Lastly, we profiled miRNA expression in N-starved rice, and identified their target genes in 7 day N-starved roots. These unprecedented large-scale, integrative analyses of the N-starved rice transcriptome will provide valuable resources for researchers developing novel gene resources for NUE-improved crop development.

We attempted to predict the potential functions of the N and Pi starvation-responsive putative RHC-class lncRNA Chr04G0017. Previous reports have indicated that short peptide-coding RNAs function as both RNAs and peptides [82–84]. To assess the potential functions of Chr04G0017 as a 116 amino acid peptide, we performed protein BLAST and peptide structure prediction. No homologous plant proteins were identified other than those in *Oryza* subspecies via protein BLAST analysis; however, weak similarities were observed with some cytoskeleton-like proteins, such as Type-II keratin, of other species. Peptide structure prediction using PEP-FOLD3 [85] showed the presence of plausible alpha-helical structure models (coiled-coil) between amino acid positions 31 and 100 (Additional file 1: Figure. S10), supporting the existence of cytoskeletal protein-like structures. Because protein BLAST results of peptide sequences from Nipponbare cultivar showed high similarities with those of other *Oryza* subspecies, we analyzed the sequence alignment of Chr04G0017 genomic region between *Oryza* subspecies. Results showed near-perfect similarities in both intronic and exonic regions of Chr04G0017, including ORF sequences (blue-colored in Additional file 1: Figure. S9), suggesting that Chr04G0017 is well-conserved among *Oryza* subspecies. Although sufficient clues could not be identified for understanding the function of Chr04G0017, sequence alignment indicated that this putative RHC-class lncRNA may play a conserved role in nutrient-deficient conditions, such as during N and Pi starvation. Further investigation is needed to understand the potential functions of both peptide and RNA forms of Chr04G0017.

Information on strand-specificity from strand-specific RNA-Seq and 2P-Seq data was helpful in defining transcriptional direction of putative lncRNAs, for identifying exact transcript models, and for measuring the expression of each transcript of *cis*-NAT pairs. This allowed us to confirm the expression of two *cis*-NAT pairs, *AMT1.1*–*cis*-NAT_*AMT1.1*_ and *AMT1.2*–*cis*-NAT_*AMT1.2*_. Moreover, we observed small RNA reads covering the overlapped region of *cis*-NAT_*AMT1*_ pairs, whereas small RNAs were not significantly detected in the overlapped region of *AMT2*–*cis*-NAT_*AMT2*_ or *AMT3*–*cis*-NAT_*AMT3*_ pairs (Additional file 1: Figure. S8). Our data suggested that these small RNAs were generated from corresponding sense transcripts rather than dsRNAs formed by complementary binding of *cis*-NAT pairs. Previous studies of the molecular functions of *cis*-NATs reported RNA-RNA interaction-based regulatory mechanisms through complementary sequence binding between *cis*-NAT pairs, leading to dsRNA-dependent RNA interference, targeted RNA protection by RNA masking and translational enhancements [21, 80]. Because these two *cis*-NAT_*AMT1*_ pairs overlapped significantly, and the nucleotide sequences of the three rice *AMT1* genes were highly similar to one other, the possibilities remain of *in cis* or *in trans* interactions between *cis*-NAT_*AMT1*_ and *AMT1* genes. Similar *cis*-NAT_*AMT1*_ transcripts also appeared to be expressed in *Arabidopsis* (At4G13505 for *AtAMT1.1 cis*-NAT pair), *Hordeum vulgare* (PUT-169a-Hordeum_vulgare-46,748), and maize (GRMZM2G474905 for *cis*-NAT_*ZmAMT1*_, GRMZM2G332891 for *cis*-NAT_*ZmAMT2*_), suggesting that *cis*-NAT_AMT_ expression and function might be conserved among plants. Further research is needed to determine the molecular functions of these *cis*-NATs in N-starved rice.

By profiling miRNA expression and analyzing Degradome sequencing datasets, we discovered a novel target of osa-miR444a.4-3p, *OsMADS25*. *OsMADS25* is one out of five *ANR1*-like rice MADS-box proteins [40, 86], all of which, except *OsMADS25*, are regulated by members of the osa-miR444 family derived from their NATs [70, 72]. Evidence from recent studies demonstrated the importance of miR444-mediated regulation of *ANR1*-like MADS-box genes in modulating N homeostasis in rice [40]. Furthermore, previous research showed that overexpression of *OsMADS25* in rice promoted primary/lateral root growth, increased shoot fresh weight, and increased nitrate accumulation in the presence of nitrate [86]. However, the relationship between the osa-miR444 family and *OsMADS25* was not noted previously as the genomic region of *OsMADS25* did not transcribe miR444-encoding *cis*-NATs. Our discoveries of N starvation-responsive characteristics of osa-miR444a.4-3p and its *in trans* regulation of *OsMADS25* expanded the regulatory pathways of the osa-miR444 family and reinforced the significance of osa-miR444 in modulating N homeostasis in rice. As a significant proportion of osa-miR444 family expression in roots (~ 47%) is attributable to osa-miR444a.4, and osa-miR444a.4 is only minimally expressed in shoots (< 0.01%), the roles of osa-miR444a.4 in N acquisition and homeostasis in roots merit further investigation.

## Conclusions

In this study, multiple transcriptomic investigations on N-starved rice plants improved our understanding of the transcriptomic responses to N starvation by providing detailed and intricate information on changes in the rice transcriptome. Strand-specific RNA-Seq datasets not only provided information on the responses of genes involved in N metabolism- and transport-involved genes, but also newly identified 2588 novel putative lncRNA encoding genomic loci. Information on the transcript models of these lncRNAs combined with the results of 2P-Seq analysis showed examples of alternatively polyadenylated isoforms of N starvation-responsive lncRNAs, providing precise information on transcript models of these lncRNAs. Analyses of lncRNAs using previously published RNA-Seq datasets revealed lncRNAs that not only responded to N starvation but also showed differential expression in response to various kinds of abiotic stresses. We also reported N-responsive characteristics of the root-specific osa-miR444a.4-3p and its novel target gene, *OsMADS25*. Overall, these large-scale datasets provide valuable information for the generation of new rice cultivars with higher NUE or greater resistance to N starvation in future breeding programs.

## Methods

### Plant material and growth conditions

Rice (*Oryza sativa* cv. Nipponbare) seeds were germinated in MS media for 4 d, and then transferred to tap water for 3d before being transferred into the hydroponic solution. Rice seedlings were grown in the modified Yoshida solution for 10 d [87]. The solution was renewed every 3 d. For preparing N-starved rice samples, seedlings were transferred to solution lacking N (0 mM of NH_4_NO_3_), and roots and shoots were harvested separately at 1, 3, 5, 7 d of N starvation (Fig. 1a). All samples were harvested at the same time of the day (i.e., 2 h after the onset of subjective day) to minimize potential circadian effects.

### Total RNA isolation and library preparation for high-throughput sequencing

Root and shoot samples were ground in liquid N, separately. Total RNA was extracted from the samples using TRIzol Reagent (Invitrogen), according to the manufacturer’s instructions, and the integrity and quality of RNA samples was analyzed. Strand-specific RNA-Seq libraries were constructed using 5 μg total RNA, according to the modified protocol previously described [88]. For poly A-primed sequencing, 2P-Seq library was constructed using 60 μg total RNA extracted from rice shoots and roots under normal and N-starvation conditions [89]. Using Illumina HiSeq 2500, strand-specific RNA-Seq libraries were analyzed with 101-bp paired-end sequencing, and 2P-Seq libraries were analyzed with 101-bp single-end sequencing. The construction and sequencing of small RNA-Seq libraries were performed according to protocols described previously [90]. The Degradome library of 7 d N-starved rice roots was constructed as described previously [91]. Degradome library were analyzed using Illumina HiSeq 2500 51-bp single-end sequencing.

### Analysis of RNA-Seq data using bioinformatics

Strand-specific RNA-Seq reads were aligned to the rice IRGSP-1.0 genome [92] using TopHat in the Cufflinks package [42], and RABT assembly was performed using Cufflinks package using the rice gene model annotation from RAP-DB (http:// http://rapdb.dna.affrc.go.jp/). Identification of putative lncRNAs was performed with analysis pipeline described in Fig. 1b. Transcript abundance of genes and putative lncRNAs was then estimated as fragments per kilobase of exon (FPKM). RNA-Seq datasets of Pi-starved rice plants were obtained from Gene Expression Omnibus (GEO), with the accession numbers SRR1005258, SRR1005300, SRR1005306, SRR1005318, SRR1005321, SRR1005363, SRR1005369, and SRR1005381 (https://www.ncbi.nlm.nih.gov/bioproject/PRJNA215013) [60], and RNA-Seq datasets of abiotic stress-treated rice plants were obtained with the accession numbers of ERR037679, ERR037681, ERR037683 and ERR037687 (https://trace.ncbi.nlm.nih.gov/Traces/study/?acc=ERP000760) [69].

For 2P-Seqdata analysis, sequence reads ranging from 20 to 95-nt were aligned to the rice genome using TopHat for further analysis. The 5′-ends of sequence reads were used to represent the genomic position of aligned 2P-Seq data, and peak signals were calculated. Peak signals up to 1000-bp distant from the 3′ end of assembled transcript models were selected as candidate polyadenylation sites.

Raw sequence reads generated from small RNA-Seq and Degradome sequencing were cleaned by removing adapter sequences. Rice miRNA prediction was performed as described previously [93]. Expression levels of miRNAs were normalized and estimated as reads per 40 million of sequence reads (RP40M). Degradome data analysis was performed to identify miRNA target genes. High-quality Degradome sequence reads were obtained from rawdata by filtering out poor quality reads and removing adapter sequences using FASTX toolkit (http://hannonlab.cshl.edu/fastx_toolkit/). Reads corresponding to structural non-coding RNAs and repeat sequences in the rice genome database [92] were filtered-out. The processed Degradome reads were analyzed using CleaveLand4 pipeline [94] with rice transcript models and miRNA sequences.

### Gene ontology (GO) analysis

GO analysis was performed using AgriGO web server with default options [95] (http://bioinfo.cau.edu.cn/agriGO/). MSU (v7.0) gene ID corresponding to differentially expressed (up- or down-regulated by more than 2-fold) RAP-DB gene ID was used in GO analysis.

### Identification of 5′ and 3′ends of transcripts and miRNA cleavage sites, and quantitative real-time-PCR (qRT-PCR)

To identify the 5′ and 3′ ends of transcripts, 5′ RNA ligase-mediated rapid amplification of cDNA ends (RLM-RACE) and 3′ RACE were performed respectively, using 1 μg total RNA and GeneRacer Kit (Invitrogen), following the manufacturer’s instructions. To identify miRNA cleavage sites in target mRNAs, 5′ RLM-RACE without serial dephosphorylation-decapping treatment was performed [96]. For quantitative RT-PCR, cDNA was synthesized from 1 μg of total RNA using oligo(dT)_18_ primers and random hexamers. Results were normalized against *UBQ5* (Os01g0328400) for shoots and *eEF-1α* (Os03g0177500) for roots. To measure the expression level of Chr04G0017, results from both shoots and roots were normalized relative to *eEF-1α*.

### Small RNA northern blot analysis

To examine the expression level of miRNAs in N-starved rice roots and shoots, 10 μg of total RNA from roots and shoots of N-starved rice samples were resolved by 15% urea-PAGE, and transferred to Hybond-NX membrane (GE Healthcare), followed by UV-crosslinking. 10 nmole of each probe corresponding to rice miRNA sequences was radiolabeled by standard 5′ end-labeling reaction using T4 polynucleotide kinase (Takara). Probe sequences used in this study were shown in Additional file 2: Table S3.

## Additional files

Additional file 1: **Figure S1.** Correlation analysis of strand-specific RNA-Seq and small RNA-Seq. **Figure S2.** GO analysis of up-regulated and down-regulated genes under nitrogen and phosphate starvation in rice. **Figure S3.** RNA-Seq read distribution of putative lncRNAs responsive to nitrogen starvation and other stressors. **Figure S4.** Expression patterns of N-responsive putative lncRNAs and quantitative PCR validation results. **Figure S5.** Predictions of miR169-targeted rice NF-YAs and 5′ RACE results. **Figure S6.** Degradome sequencing analysis of genes targeted by rice microRNAs. **Figure S7.** RNA-Seq read distribution on the genomic region of ammonium transporters, and predicted secondary structures. **Figure S8.** RNA-Seq and Small RNA-Seq read distribution on the genomic region of AMT2.1 and AMT3.3. **Figure S9.** Sequence alignment of Chr04G0017 genomic region in rice subspecies. Blue characters indicate predicted open reading frame. **Figure S10.** Peptide modeling results of Chr04G0017-encoding open reading frame. (PDF 33992 kb)

Additional file 2: **Table S1.** Read statistics of the four types of transcriptome data used in this study. **Table S2.** List of genes involved in nitrogen source uptake, assimilation and transport and their expression level in rice. **Table S3.** List of amino acid/peptide transporter genes and their expression level in rice. **Table S4.** Pipeline for identification of putative long non-coding RNAs in rice. **Table S5.** List of putative long non-coding RNAs identified in this study and their expression level in rice. **Table S6.** List of miRNAs analyzed in this study and their expression level in rice. **Table S7.** List of microRNA-targeted genes identified by Degradome-Seq. **Table S8.** Oligonucleotide sequences used in this study. (XLSX 1030 kb)

## Abbreviations

2P-Seq: Poly A-primed sequencing
AAP: Amino acid permease
AAT: Amino acid transporter
AMT: Ammonium transporter
ASNS: Asparagine synthetase
GOGAT: Glutamine-oxoglutarate aminotransferase
GS: Glutamine synthetase
IRGSP: International Rice Genome Sequencing Project
LncRNA: Long non-coding RNA
MADS: MCM1/AGAMOUS/DEFICIENS/SRF
NAT: Natural antisense transcript
NF-YA: Nuclear transcription factor Y subunit alpha
NiR: Nitrite reductase
NR: Nitrate reductase
NRT: Nitrate transporter
NUE: Nitrogen Use Efficiency
OPT: Oligopeptide transporter
PTR: Peptide transporter
qRT-PCR: quantitative real-time PCR
RABT: Reference annotation-based transcript
RAP-DB: Rice Annotation Project Database
