# Supplementary material for: Transcriptomic analyses of rice (Oryza sativa) genes and non-coding RNAs under nitrogen starvation using multiple omics technologies
Source: BMC Genomics. 2018 Jul 13;19:532. doi: 10.1186/s12864-018-4897-1 (PMC6043990; doi:10.1186/s12864-018-4897-1)

Figure S1. Correleation analysis of strand-specific RNA-Seq and small RNA-Seq

(A) Correleation plot for strand-specific RNA-Seq results.
Expression levels of genes are estimated as fragments per kilobase of exon per million reads (FPKM).

(B) Correleation plot for small RNA-Seq results.
Expression levels of sequenced small RNAs (including microRNAs) are estimated as reads per 40 million of sequenced reads (RP40M)

A

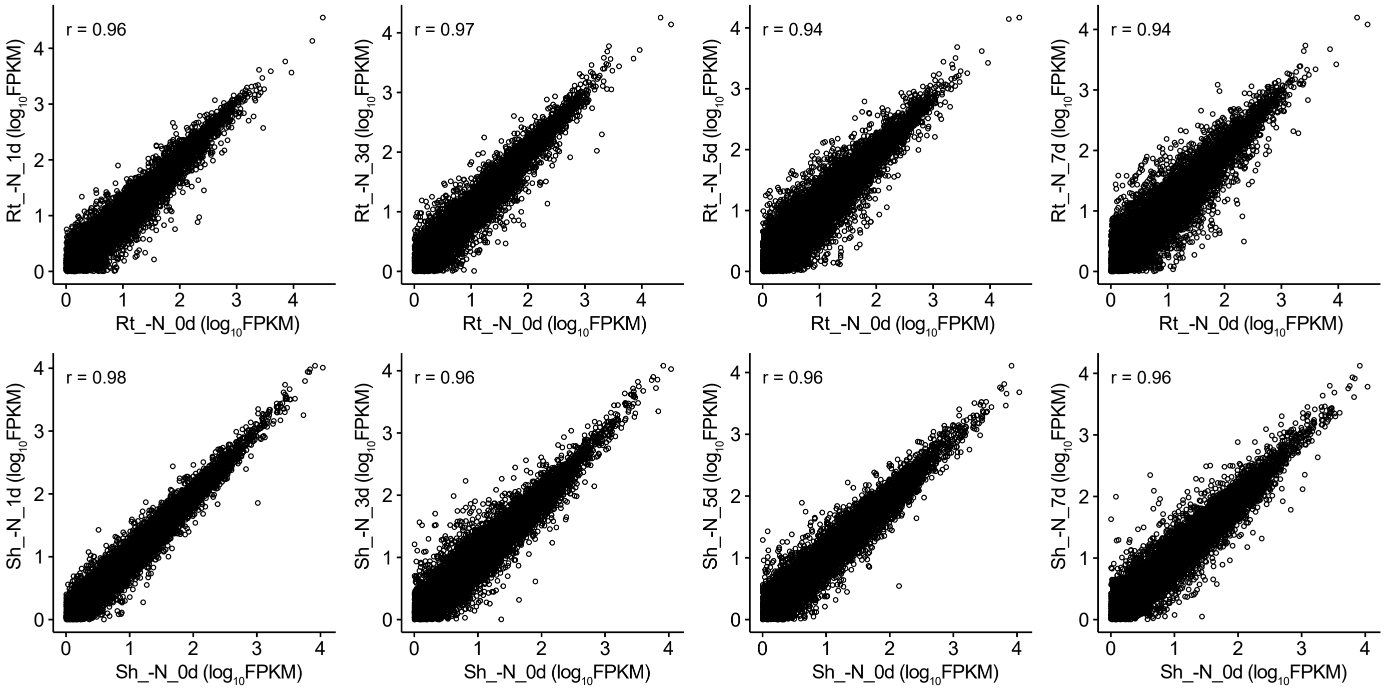

B

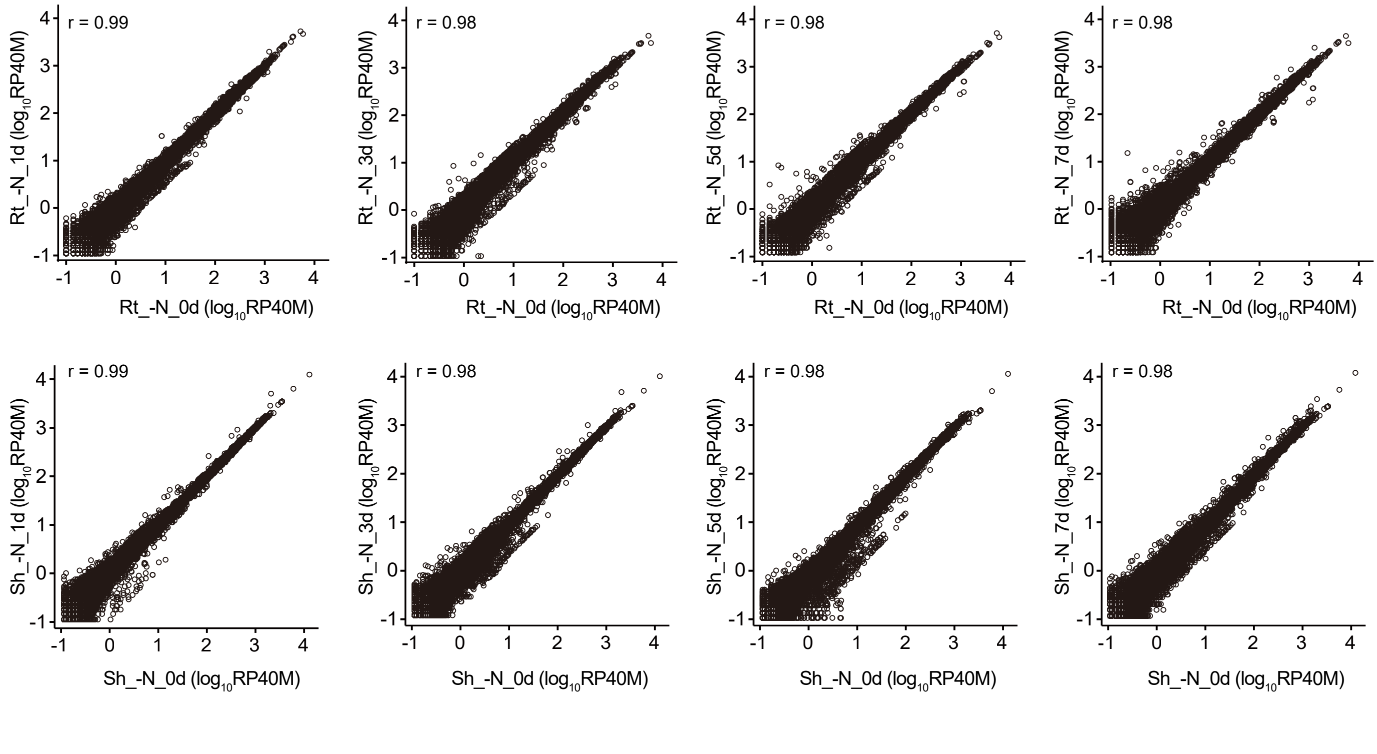

Figure S2. GO analysis of up-regulated and down-regulated genes under nitrogen and phosphate starvation in rice.

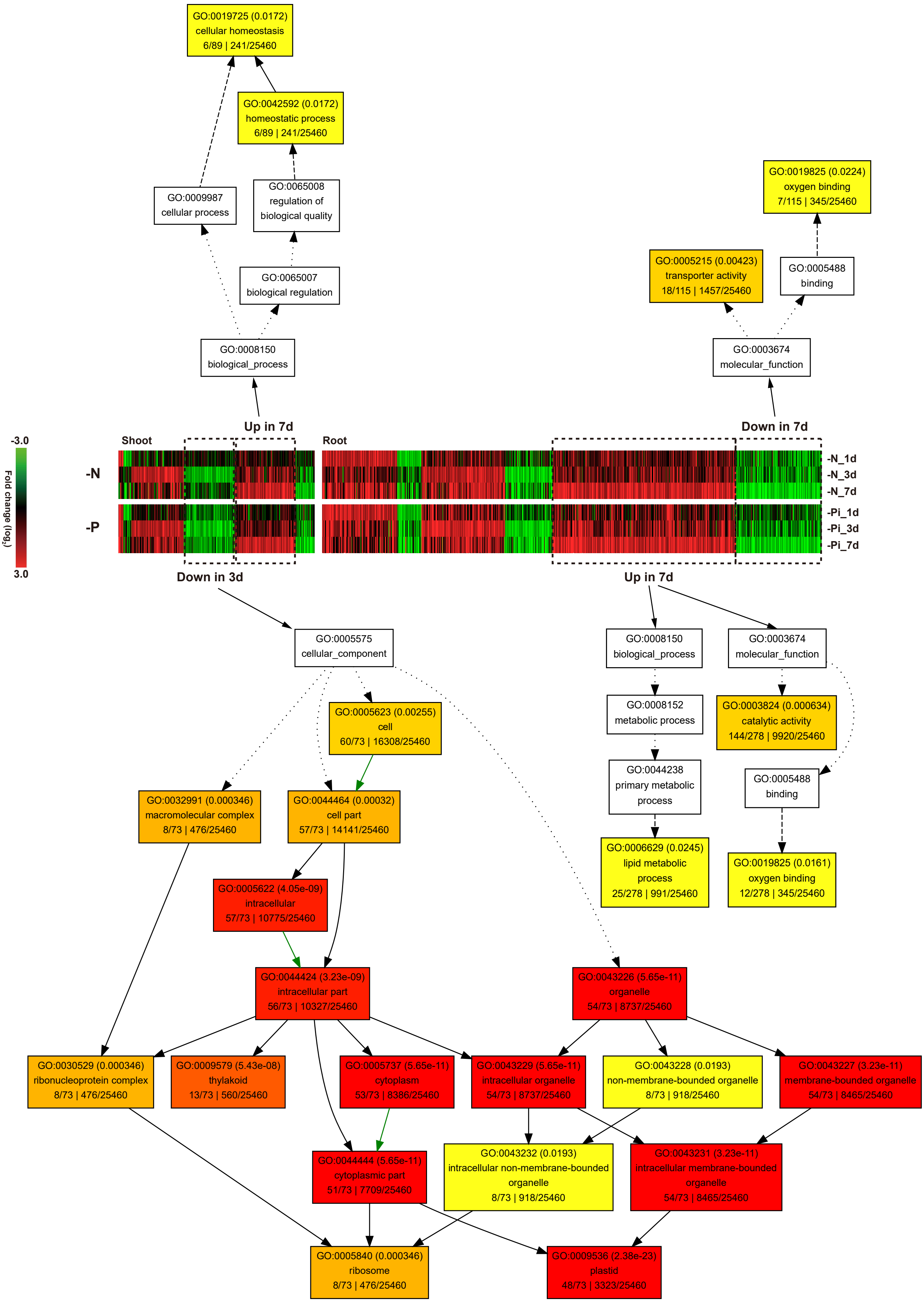

Figure S3. RNA-Seq read distribution of putative lncRNAs responsive to nitrogen starvation and other stressors.
(A) RNA-Seq read distribution of nitrogen starvation and cold stress-responsive putative long non-coding RNA, Chr03G0008.
(B) RNA-Seq read distribution of nitrogen and phosphate starvation-responsive putative long non-coding RNA, Chr07G0116.

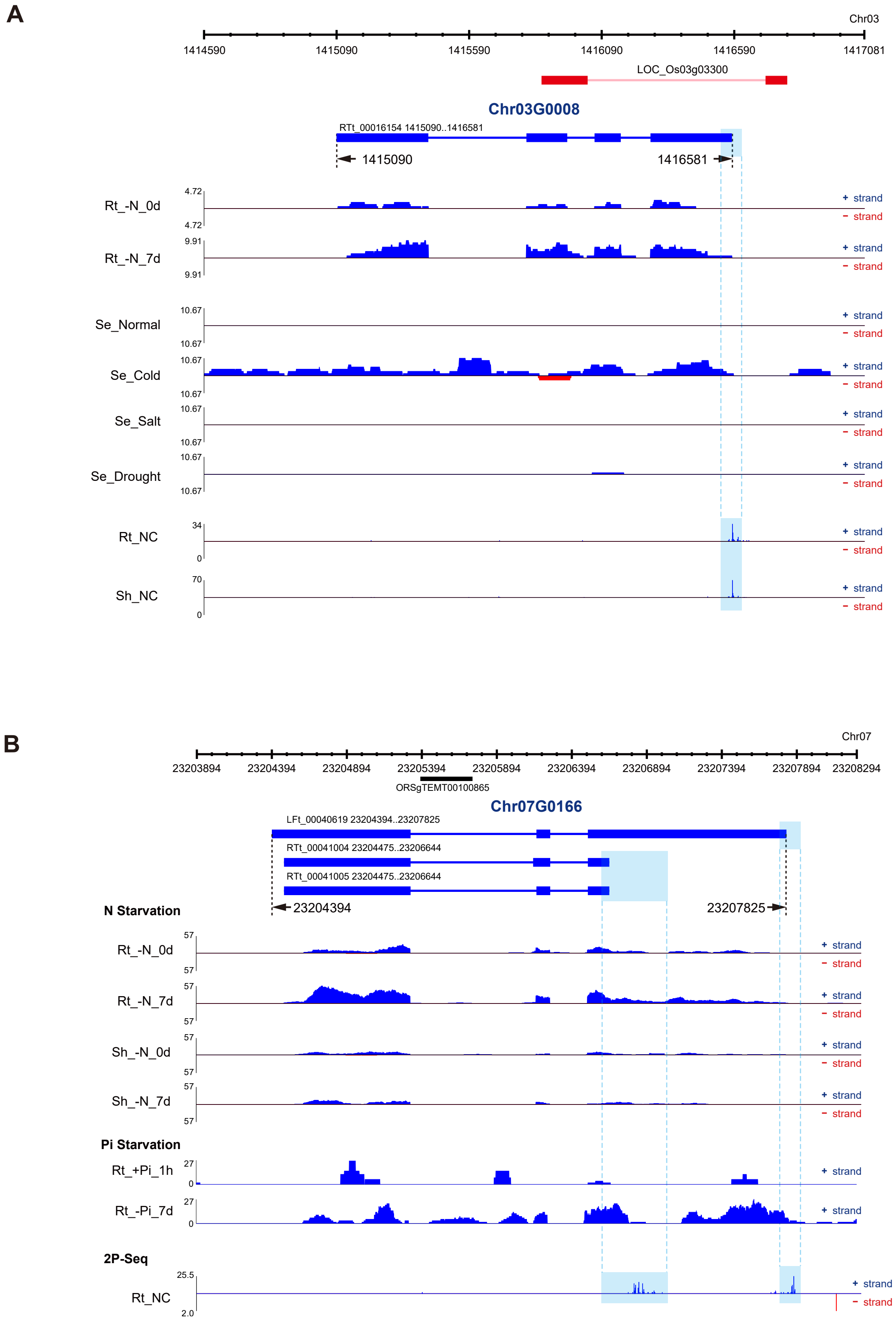

Figure S4. Expression patterns of N-responsive putative lncRNAs and quantitative PCR validation results.
(A) Chr01G0100, (B) Chr03G0086, (C) Chr04G0017, (D) Chr04G0169, (E) Chr07G0166, (F) Chr11G0185,
N.D.; not detected in quantitative PCR results

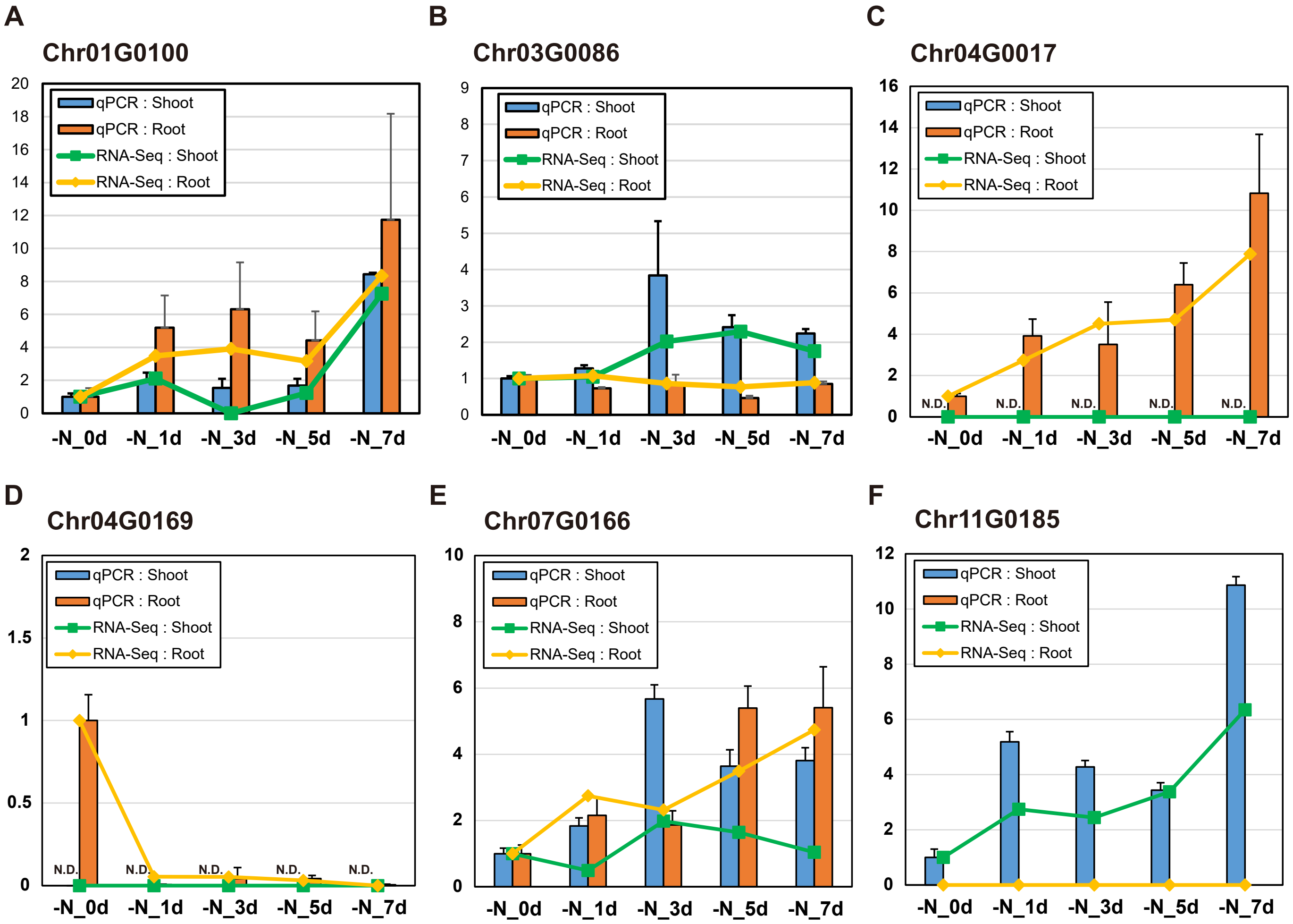

Figure S5. Predictions of miR169-targeted rice NF-YAs and 5′ RACE results.
(A) Transcript structure and miR169-binding sites of rice NF-YAs, and target pairing score of each miR169 family members.
(B) Gel electrophoresis results of NF-YA 5′ RACE product. Red arrows indicate predicted product size of NF-YA transcript cleaved by miR169 family members.
(C) to (I) Sequencing results of NF-YA 5′ RACE product. Red arrows indicate predicted miR169-cleaving site of each NF-YA.
Numbers on red arrow indicates predicted cleavage site-aligned reads per total sequenced clone numbers.

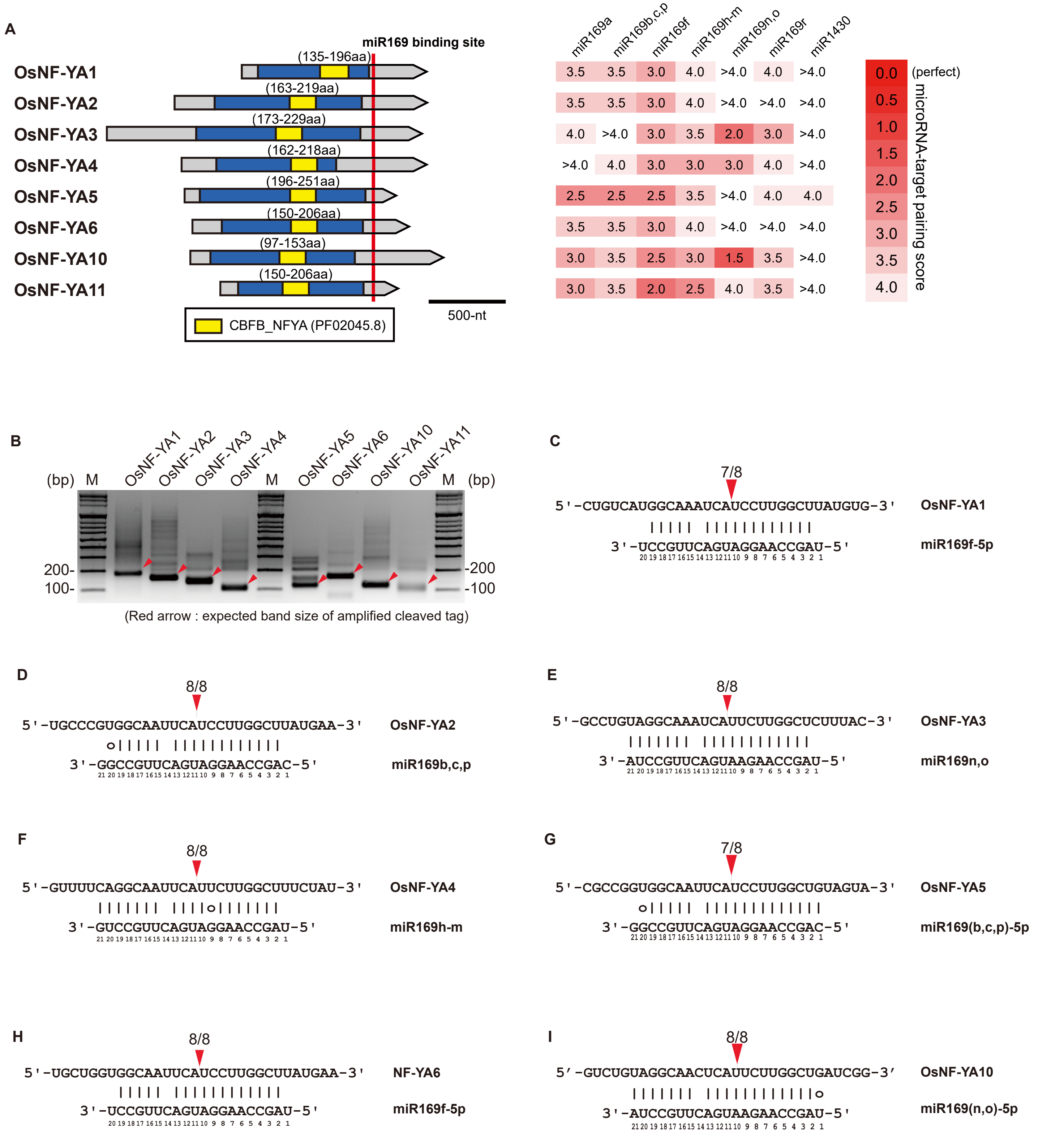

Figure S6. Degradome sequencing analysis of genes targeted by rice microRNAs.

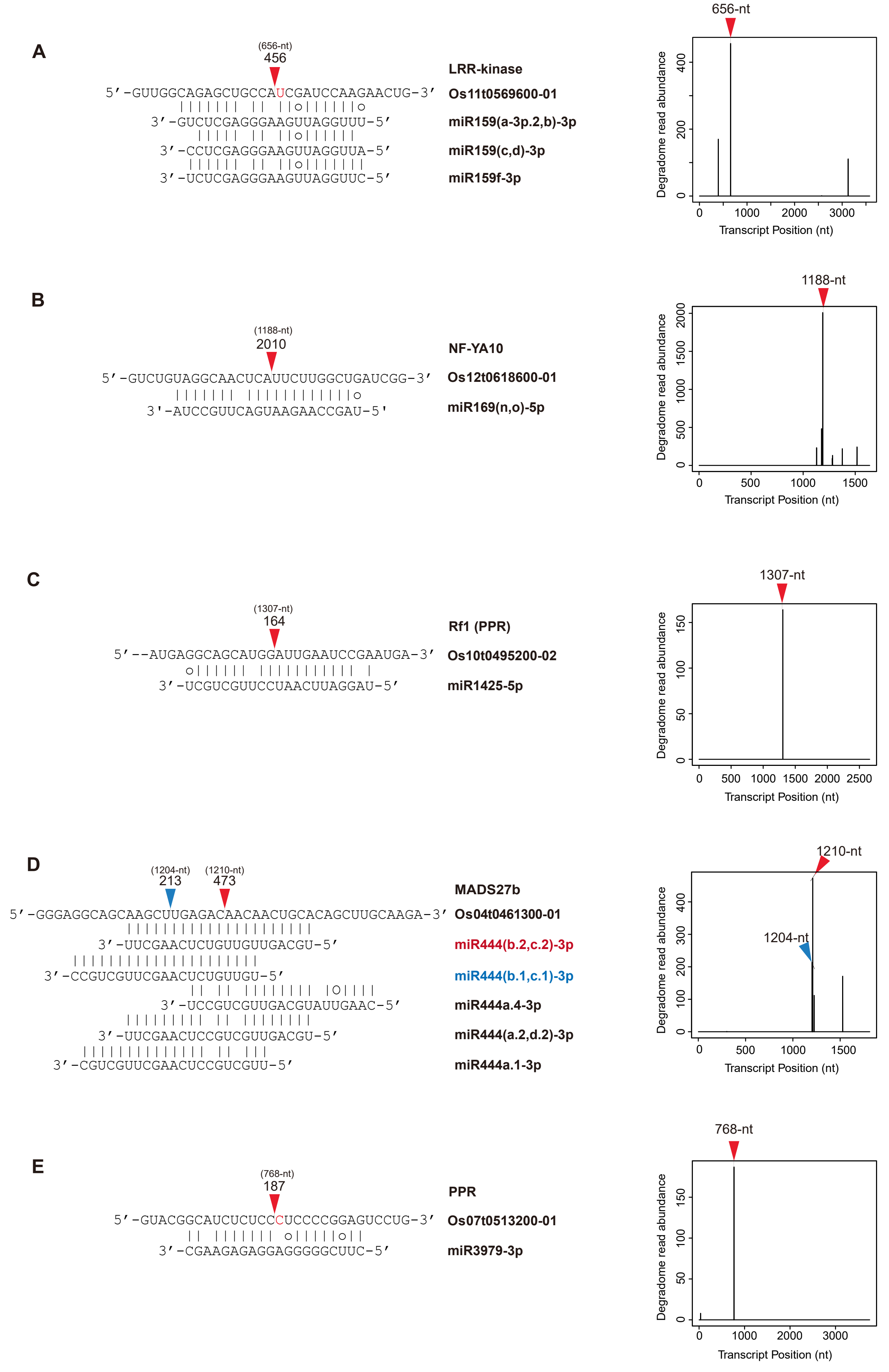

Figure S7. RNA-Seq read distribution on the genomic region of ammonium transporters, and predicted secondary structures. (A) Read distribution on genomic region of *AMT1.2* and *cis*-NAT$_{AMT1.2}$. (B) (C) RNA-FOLD prediction results of *AMT1.1* and *AMT1.2* transcripts.

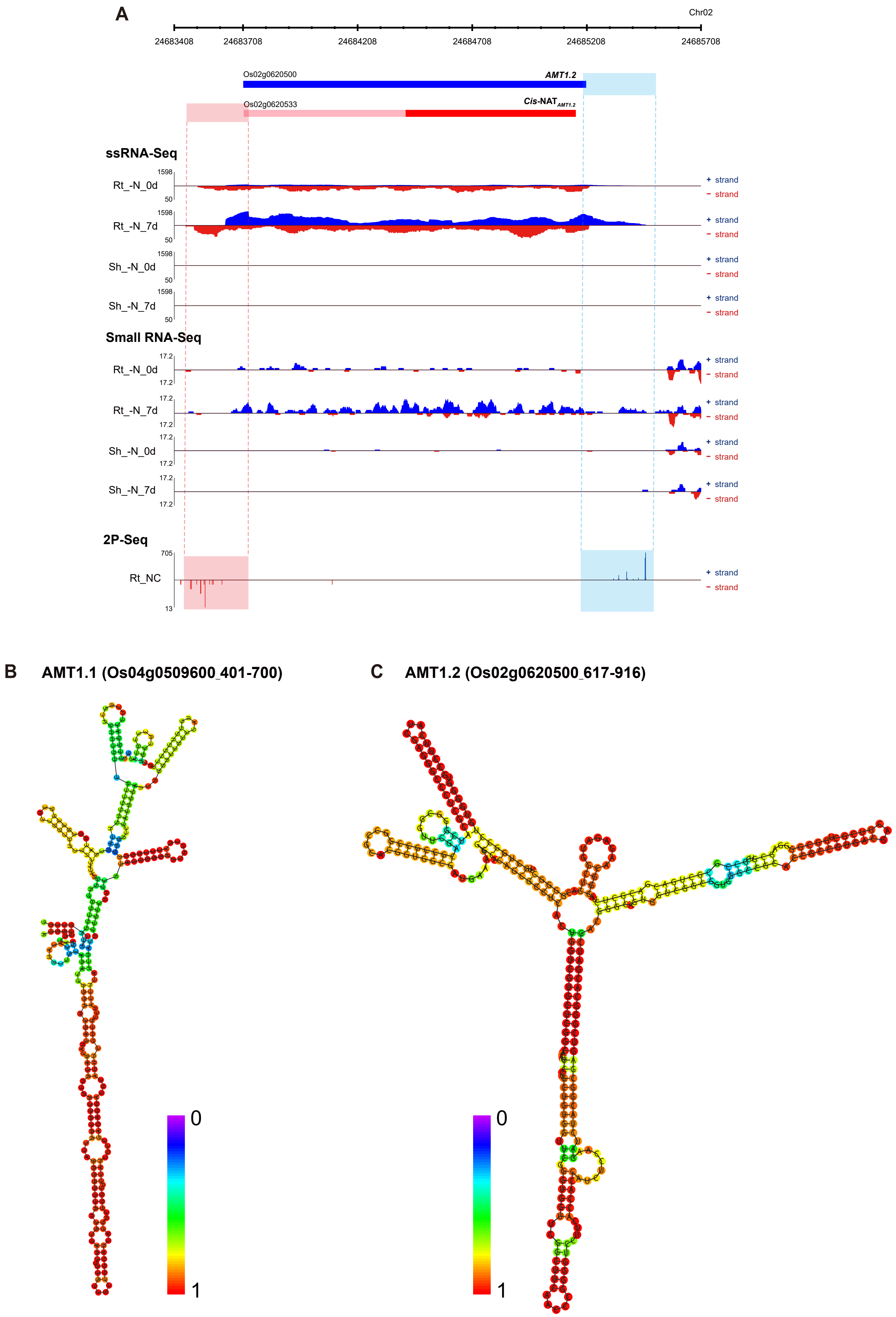

Figure S8. RNA-Seq and Small RNA-Seq read distribution on the genomic region of *AMT2.1* and *AMT3.3*.

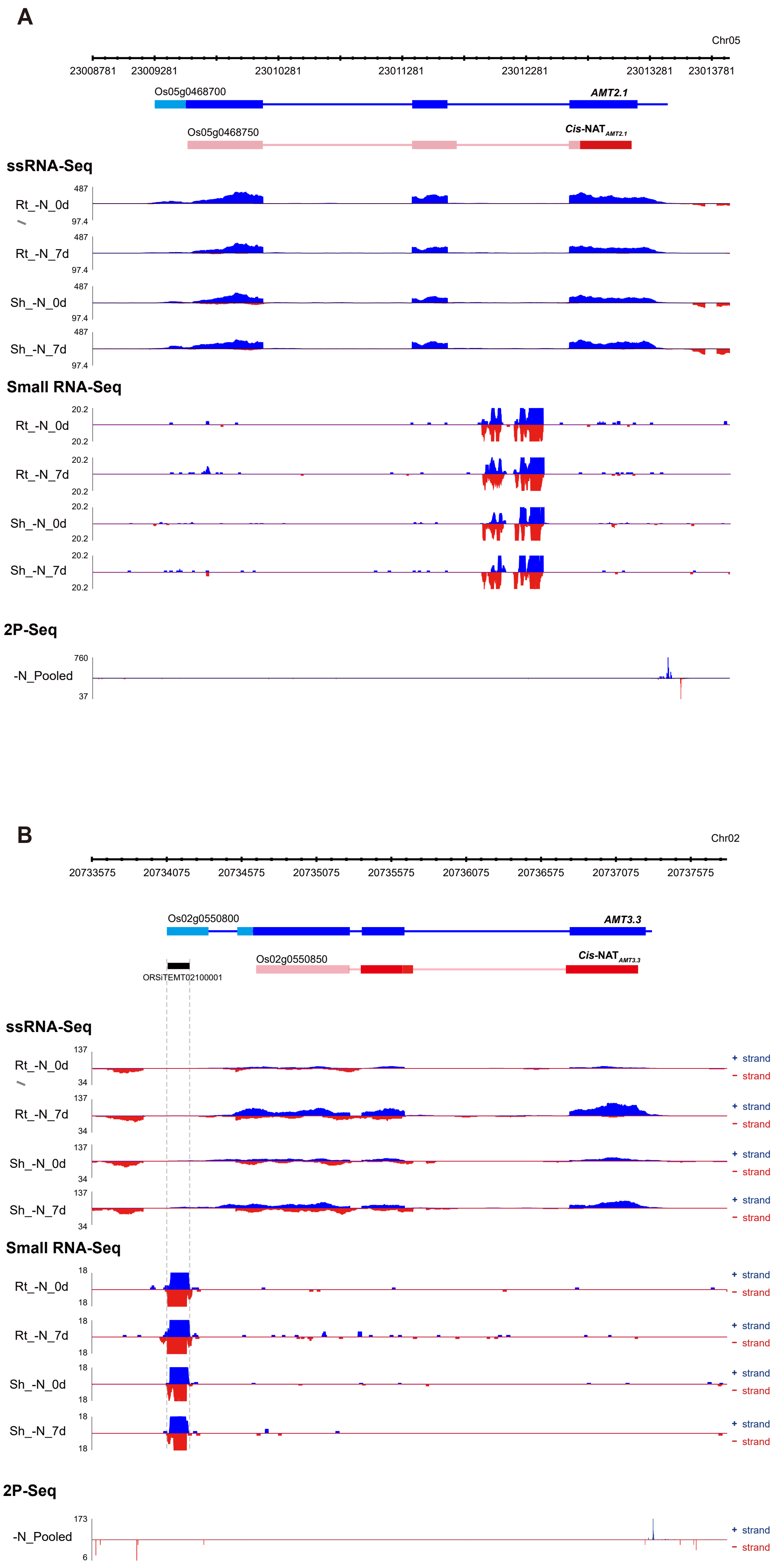

Figure S9. Sequence alignment of Chr04G0017 genomic region in rice subspecies.

Blue characters indicate predicted open reading frame. Upper and lower characters represent exon and intron regions of Chr04G0017, respectively.

```
                                                                                          Start codon
Chr04G0017__RTt_00023683     ACCAAGGTTAGAATAACTCAAGGATCAAGTTTGCAACTGATCGAATTTGCTAGCTAGCTGGTTTCACAATGgtactgatcttctcttgctatacgcatat
93-11_chr04                  ACCAAGGTTAGAATAACTCAAGGATCAAGTTTGCAACTGATCGAATTTGCTAGCTAGCTGGTTTCACAATGgtactgatcttctcttgctatacgcatat
Pa64_chr04                   ACCAAGGTTAGAATAACTCAAGGATCAAGTTTGCAACTGATCGAATTTGCTAGCTAGCTGGTTTCACAATGgtactgatcttctcttgctatacgcatat
Oryza_barthii_chr04          ACCAAGGTTAGAATAACTCAAGGATCAAGTTTGCAACTGATCGAATTTGCTAGCTAGCTGGTTTCACAATGgtactgatcttctcttgctatacgcatat
Oryza_glaberrima_chr04       ACCAAGGTTAGAATAACTCAAGGATCAAGTTTGCAACTGATCGAATTTGCTAGCTAGCTGGTTTCACAATGgtactgatcttctcttgctatacgcatat
Oryza_meridionalis_chr04     ACCAAGGTTAGAATAACTCAAGGATCAAGTTTGCAACTGATCGAATTTGCTAGCTAGCTGGTTTCACAATGgtactgatcttctcttgctatacgcatat
Oryza_nivara_chr04           ACCAAGGTTAGAATAACTCAAGGATCAAGTTTGCAACTGATCGAATTTGCTAGCTAGCTGGTTTCACAATGgtactgatcttctcttgctatacgcatat

Chr04G0017__RTt_00023683     actactatgatcttcgtcccaaaatatagttactttaaacgtttaaatttattttttttcttatattacttgtctacctagctatttcttatatatct
93-11_chr04                  actattatgatcatcgtcccaaaatatagttactttaagcgtttaaatttattttttttcttatattacttgtctacctagctatttcttatatatct
Pa64_chr04                   actactatgatcttcgtcccaaaatatagttactttaaacgtttaaatttattttttttcttatattacttgtctacctagctatttcttatatatct
Oryza_barthii_chr04          actactacgatcttcgtcccaaaatatagttactttaaacgtttaaatttattttttt-cttatattacttgtctacctagctatttcttatatatct
Oryza_glaberrima_chr04       actactacgatcttcgtcccaaaatatagttactttaaacgtttaaatttattttttt-cttatattacttgtctacctagctatttcttatatatct
Oryza_meridionalis_chr04     actactacgatcttcgttccaaaatatagttactttaagcatttaaatttattttttt-cttatattacttgtctacctatctatttcttatatatct
Oryza_nivara_chr04           actattatgatcttcgtcc----------ttactttaagcgtttaaatttattttttttcttatattacttgtctacctagctatttcttatatatct

Chr04G0017__RTt_00023683     catcatttgtttagtttctccaccagctgcttgtccctaccaaagtaccaatcattactaattaattttcttttaatgttacgtctattcccaatcctt
93-11_chr04                  catcatttgtttagtttctccaccagctgcttgtccctaccaaagtaccaatcattactaattaattttcttttaatgttacgtctattcccaatcctt
Pa64_chr04                   catcatttgtttagtttctccaccagctgcttgtccctaccaaagtaccaatcattactaattaattttcttttaatgttacgtctattcccaatcctt
Oryza_barthii_chr04          catcatttgtttagtttctccaccagctgcttgtccctaccaaagtaccaatcattactaattaattttcttttaatgttacgtctattcccaatcctt
Oryza_glaberrima_chr04       catcatttgtttagtttctccaccagctgcttgtccctaccaaagtaccaatcattactaattaattttcttttaatgttacgtctattcccaatcctt
Oryza_meridionalis_chr04     catcatttgtttagtttctccaccagctgcttgtccctactaaagtactaatcattactaattaattttcttttaatgttacgtctattcccaatcctt
Oryza_nivara_chr04           catcatttgtttagtttctccaccagctgcttgtccctaccaaagtaccaatcattactaattaattttcttttaatgttacgtctattcccaatcctt

Chr04G0017__RTt_00023683     gtaatgaaataactcgaccgatgaaggaaagaataagaaaaagcttcaaccaaactggctaaaaatggccacgaactttggcccatacatgcggattagt
93-11_chr04                  gtaatgaaataactcgaccgatgaaggaaagaataagaaaaagcttcaaccaaactggctaaaaaggccacgaactttggcccatacatgcggactagt
Pa64_chr04                   gtaatgaaataactcgaccgatgaaggaaagaataagaaaaagcttcaaccaaactggctaaaaatggccacgaactttggcccatacatgcggattagt
Oryza_barthii_chr04          gtaatgaaataactcgaccgatgaaggaaagaataagaaaaagcttcaaccaaattggctaaaaaggccacgaactttggcccatacacgcggactagt
Oryza_glaberrima_chr04       gtaatgaaataactcgaccgatgaaggaaagaataagaaaaagcttcaaccaaattggctaaaaaggccacgaactttggcccatacacgcggactagt
Oryza_meridionalis_chr04     gtaatgaaataactcgactgatgaaggaaagaataagaaaaagcttcaaccaaactggctaaaaaggccacgaactttggcccatacttgcggactagt
Oryza_nivara_chr04           gtaatgaaataactcgaccgatgaaggaaagaataagaaaaagcttcaaccaaactggctaaaaaggccacgaactttggcccatacatgcggactagt

Chr04G0017__RTt_00023683     ccttatgatctgtcattaacccatgttttaagacctctgtctttcagatgatgggcccattcttaatacacgtttttacgaggagtctacaagtgatctt
93-11_chr04                  ccttatgatctgtcattaacccatgttttaagacctctgtcttttagatgatgggcccattcttaatacacgtttttactaggagtctacaagtgatctt
Pa64_chr04                   ccttatgatctgtcattaacccatgttttaagacctctgtctttcagatgatgggcccattcttaatacacgtttttacgaggagtctacaagtgatctt
Oryza_barthii_chr04          ccttaagatctttcattaacccatgttttaagacctctgtctttcagatgatgggcccattcttaatatacgtttttacgaggagtctacaagtgatctt
Oryza_glaberrima_chr04       ccttaagatctttcattaacccatgttttaagacctctgtctttcagatgatgggcccattcttaatatacgtttttacgaggagtctacaagtgatctt
Oryza_meridionalis_chr04     ccttatgatctgtcattaacccatgttttaagacctctgtctttcagatgatgggcccattcttaatacatgtttttacgaggagtctacaaatggtctt
Oryza_nivara_chr04           ccttatgatctgtcattaacccatgttttaagacctctgtctttcagatgatgggcccattcttaatacacgtttttactaggagtctacaagtgatctt

Chr04G0017__RTt_00023683     ttttaacctgaaattcgcttacatagatagtcatacccagtactactcgagcatttcaaccactaggctacgagttctttctcgcttaatccttatacaa
93-11_chr04                  ttttaacctgaaattcgcttacatagatagtcatacc------------------------actaggctacgagttctttctcgcttaatccttatacaa
Pa64_chr04                   ttttaacctgaaattcgcttacatagatagtcatacccagtactactcgagcatttcaaccactaggctacgagttctttctcgcttaatccttatacaa
Oryza_barthii_chr04          ttttaacctgaaattcgcttacatagatagtcatacccagtactactcgagcatttcaaccactaggctacgagttctttctcgcttaatccttatacaa
Oryza_glaberrima_chr04       ttttaacctgaaattcgcttacatagatagtcatacccagtactactcgagcatttcaaccactaggctacgagttctttctcgcttaatccttatacaa
Oryza_meridionalis_chr04     ttttaacctgaaattcgcttacatagatagtcatacccagtactact--------------actaggctacgagttctttctcgcttaatccttatacaa
Oryza_nivara_chr04           ttttaacctgaaattcgcttacatagatagtcatacc------------------------actaggctacgagttctttctcgcttaatccttatacaa

Chr04G0017__RTt_00023683     gatatgcaaagtgctttattgggacggatggagtgcttatatactccttccatctcaaatatagcattagattcatcgtatatctggatatggtctcacc
93-11_chr04                  gatatgcaaagtgctttattgggacggatggagtgcttatatactccttccatctcaaatatagcattagattcatcgtatgtctggatatggtctcacc
Pa64_chr04                   gatatgcaaagtgctttattgggacggatggagtgcttatatactccttccatctcaaatatagcattagattcatcgtatatctggatatggtctcacc
Oryza_barthii_chr04          gatatgcaaagtgctttattgggacggatgaagtgcttatatactccttccatctcaaatatagcattagattcatcgtatatctggatatggtctcacc
Oryza_glaberrima_chr04       gatatgcaaagtgctttattgggacggatgaagtgcttatatactccttccatctcaaatatagtattagattcatcgtatatctggatatggtctcacc
Oryza_meridionalis_chr04     gatatgcaaagtgctttattgggacggatggagtgcttatatactccttccatctcaaatatagcattagattcatcgtatatctggatatggtctcacc
Oryza_nivara_chr04           gatatgcaaagtgctttattggaacggatggagtgcttatatactccttccatctcaaatatagcattagattcatcgtatgtctggatatggtctcacc

Chr04G0017__RTt_00023683     cgattttagattcccatattttgtaatggagttaatattcttatcaaacatgcatatgattattatgatgatctatatatgcatgcagCACCGGGCAACA
93-11_chr04                  cgattttagattcccatattttgtaatggagttaatattcttatcaaacatgcatatgattattatgatgatctatatatgcatgcagCACCGGGCAATA
Pa64_chr04                   cgattttagattcccatattttgtaatggagttaatattcttatcaaacatgcatatgattattatgatgatctatatatgcatgcagCACCGGGCAACA
Oryza_barthii_chr04          cgattttagattcccatattttgtaatggagttaatattcttatcaaacatgcatatgattattatgatgatctatatatgcatgcagCACCGGGCAACA
Oryza_glaberrima_chr04       cgattttagattcccatattttgtaatggagttaatattcttatcaaacatgcatatgattattatgatgatctatatatgcatgcagCACCGGGCAACA
Oryza_meridionalis_chr04     cgattttagattcccatattttgtaatggagttaatattcttatcaaacatgcatatgattattatgatgatctatatatgcatgcagCACCGGGCAATA
Oryza_nivara_chr04           cgattttagattcccatattttgtaatggagttaatattcgtatcaaacatgcatatgattattatgatgatctatatatgcatgcagCACCGGGCAATA

Chr04G0017__RTt_00023683     AGAAGGGGATATACGGGCGATCCGCCAACAAAGGTACTAGAGAGAGAGAACGCCGATGCGCACAAGGATCAAGTCAAGGGCTTAAACGGCAGAATCAGTA
93-11_chr04                  AGAAGGGGATATACGGGCGATCCGCCAACAAAGGTACTAGACAGAGAGAACGCCGATGCGCACAAGGATCATGTCAAGGGCTTAAACGGCAGAATCAGTA
Pa64_chr04                   AGAAGGGGATATACGGGCGATCCGCCAACAAAGGTACTAGAGAGAGAGAACGCCGATGCGCACAAGGATCAAGTCAAGGGCTTAAACGGCAGAATCAGTA
Oryza_barthii_chr04          AGAAGGGGATATACGGGCGATCCGCCAACAACGGTACTAGAGAGAGAGAACGCCGATGCGCACAAGGATCAAGTCAAGGGCTTAAACGGCAGAATCAGTA
Oryza_glaberrima_chr04       AGAAGGGGATATACGGGCGATCCGCCAACAACGGTACTAGAGAGAGAGAACGCCGATGCGCACAAGGATCAAGTCAAGGGCTTAAACGGCAGAATCAGTA
Oryza_meridionalis_chr04     AGAAGGGGATATACGGGCGATCCGCCAACAGCGGTACTAGAGAGAGAGAACGCCGATGCGCACAAGGATCAAGTCAAGGGCTTAAACGGCAGAATCAGTA
Oryza_nivara_chr04           AGAAGGGGATATACGGGCGATCCGCCAACAAAGGTACTAGAGAGAGAGAACGCCGATGCGCACAAGGATCAAGTCAAGGGCTTAAACGGCAGAATCAGTA

Chr04G0017__RTt_00023683     AATTAAACGACACAATAAAAGAGTTAAACGACACGATCGAGGCGCTGGAGAGGCAGGTTCAGAACCTGACCAGGTACAAGGAAGAGAAGCAGAAGCAGCA
93-11_chr04                  AATTAAACGACACAATAAAAGAGTTAAACGACACGATCGAGGCGCTGGAGAGGCAGGTTCAGAACCTGACCAGGTACAAGGAAGAGAAGCAGAAGCAGCA
Pa64_chr04                   AATTAAACGACACAATAAAAGAGTTAAACGACACGATCGAGGCGCTGGAGAGGCAGGTTCAGAACCTGACCAGGTACAAGGAAGAGAAGCAGAAGCAGCA
Oryza_barthii_chr04          AATTAAACGGCACAATAAAAGAGTTAAACGACACGATCGAGGCGCTGGAGAGGCAGGTTCAGAACCTGACCAGGTACAAGGAAGAGAAGCAGAAGCGGCA
Oryza_glaberrima_chr04       AATTAAACGGCACAATAAAAGAGTTAAACGACACGATCGAGGCGCTGGAGAGGCAGGTTCAGAACCTGACCAGGTACAAGGAAGAGAAGCAGAAGCGGCA
Oryza_meridionalis_chr04     AATTAAACGGCACAATAAAAGAGTTAAACGACACGATCGAGGCGCTGGAGAGGCAGGTTCAGAACCTGACCAGGTACAAGGAAGAGAAGCAGAAGCAGCA
Oryza_nivara_chr04           AATTAAACGACACAATAAAAGAGTTAAACGACACGATCGAGGCGCTGGAGAGGCAGGTTCAGAACCTGACCAGGTACAAGGAAGAGAAGCAGAAGCAGCA

Chr04G0017__RTt_00023683     CGCGAATCTTCAGAAAGAATTCGCAGAGCTGGAGAGGAAATACAGAGACCTGGATGCAGCCCACAGGAATTGCGGCCCCACTGTCAGATTCCCTGTTTTC
93-11_chr04                  CGCGAATCTTCAGAAAGAATTCGCAGAGCTGGAGAGGAAATACAGAGACCTGGATGCAGCCCACAGGAATTGCGGCCCCACTGTCAGATTCCCTGTTTTC
Pa64_chr04                   CGCGAATCTTCAGAAAGAATTCGCAGAGCTGGAGAGGAAATACAGAGACCTGGATGCAGCCCACAGGAATTGCGGCCCCACTGTCAGATTCCCTGTTTTC
Oryza_barthii_chr04          CGCGAATCTTCAGAAAGAATTCGCAGAGCTGGAGAGGAAATACAGAGACCTGGATGCAGCCCACAAGAATTGCGGCCCCACTGTCAGATTCCCTGTTTTC
Oryza_glaberrima_chr04       CGCGAATCTTCAGAAAGAATTCGCAGAGCTGGAGAGGAAATACAGAGACCTGGATGCAGCCCACAAGAATTGCGGCCCCACTGTCAGATTCCCTGTTTTC
Oryza_meridionalis_chr04     CGCGAATCTTCAGAAAGAATTCGCAGAGCTGGAGAGGAAATACAGAGACCTGGATGCAGCCCACAAGAATTGCGGCCCCACTGTCAGATTCCCTGTTTTC
Oryza_nivara_chr04           CGCGAATCTTCAGAAAGAATTCGCAGAGCTGGAGAGGAAATACAGAGACCTGGATGCAGCCCACAGGAATTGCGGCCCCACTGTCAGATTCCCTGTTTTC
                                                       Stop codon
Chr04G0017__RTt_00023683     ACCGTCGGACAACCCTACTATCACCGCCACAATTGATCGATCGATATGATCGATC--TGCACAACCATCGAATGCCCTACGTACGTACCTGAATATATGT
93-11_chr04                  ACCGTCGGACAACCCTACTATCACCGCCACAATTGATCGATCGATATGATCGATC--TGCACAACCATCGAATGCACTACGTACGTACCTGAATATATGT
Pa64_chr04                   ACCGTCGGACAACCCTACTATCACCGCCACAATTGATCGATCGATATGATCGATC--TGCACAACCATCGAATGCCCTACGTACGTACCTGAATATATGT
Oryza_barthii_chr04          ACCGTCGGACAACCCTACTATCACCACCACTATTGATCGATCTGCACAACCATCGAATGCACTACGTACGTAC-CTGAATATATATATATATATATATGT
Oryza_glaberrima_chr04       ACCGTCGGACAACCCTACTATCACCACCACTATTGATCGATCTGCACAACCATCGAATGCACTACGTACGTAC-----CTGAATATATATATATATATGT
Oryza_meridionalis_chr04     ACCGTCGGACAACCCTACTATCACCACCACTATTGATCGATCGATATGATCGATC--TGCACAACCATCGAATGCACTACGTACGTACCTGAATATATGT
Oryza_nivara_chr04           ACCGTCGGACAACCCTACTATCACCGCCACAATTGATCGATCGATATGATCGATC--TGCACAACCATCGAATGCACTACGTACGTACCTGAATATATGT

Chr04G0017__RTt_00023683     ATAAGTATCTATACAGTATATGCAGGGTTGTGTGTGATGTATATGCATGCCTATGAGAGTTGCGAGTGTACTGTATGTATATGTGTGTGAAGAATAAAGC
93-11_chr04                  ATAAGTATCTATACAGTATATGCAGGGTTGTGTGTGATGTATATGCATGCCTATGAGAGATGCGAGTGTACTGTATGTATATGTGTGTGAAGAATAAAGC
Pa64_chr04                   ATAAGTATCTATACAGTATATGCAGGGTTGTGTGTGATGTATATGCATGCCTATGAGAGTTGCGAGTGTACTGTATGTATATGTGTGTGAAGAATAAAGC
Oryza_barthii_chr04          ATAAGTATCTATACAGTATATGCAGGGTTGTGTGTGATGTATATGCATGCCTATGAGAGTTGCGAGTGTGCTATATGTATATGTGTGTGAAGAATAAAGC
Oryza_glaberrima_chr04       ATAAGTATCTATACAGTATATGCAGGGTTGTGTGTGATGTATATGCATGCCTATGAGAGTTGCGAGTGTGCTATATGTATATGTGTGTGAAGAATAAAGC
Oryza_meridionalis_chr04     ATAAGTATCTATACAGTATATGCAGGGTTGTGTGTGATGTATATGCATGCCTATGAGAGTTGCGAGTGTACTGTATGTATATGTGTGTGAAGAATAAAGC
Oryza_nivara_chr04           ATAAGTATCTATACAGTATATGCAGGGTTGTGTGTGATGTATATGCATGCCTATGAGAGTTGCGAGTGTACTGTATGTATATGTGTGTGAAGAATAAAGC

Chr04G0017__RTt_00023683     TGCATGTATACTGGCAGCTATATATCTAGAGCAT
93-11_chr04                  TGCATGTATACTGGCAGCTATATATCTAGAGCAT
Pa64_chr04                   TGCATGTATACTGGCAGCTATATATCTAGAGCAT
Oryza_barthii_chr04          TGCATGCATACTGGCAGCTATATATCTAGAGCAT
Oryza_glaberrima_chr04       TGCATGCATACTGGCAGCTATATATCTAGAGCAT
Oryza_meridionalis_chr04     TGCATGTATACTGGCAGCTATATATCTAGAGCAT
Oryza_nivara_chr04           TGCATGTATACTGGCAGCTATATATCTAGAGCAT
```

Figure S10. Peptide modeling results of Chr04G0017-encoding open reading frame.

Chr04G0017_ORF:30-80

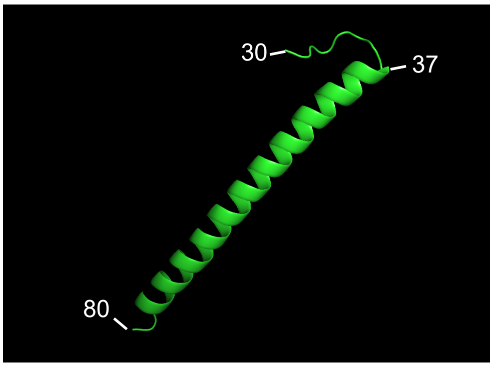

Chr04G0017_ORF:40-90

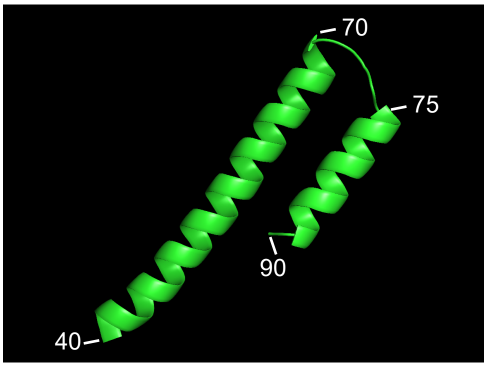

Chr04G0017_ORF:50-100

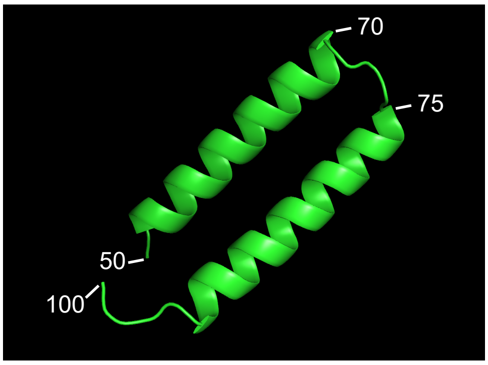

Chr04G0017_ORF:60-110

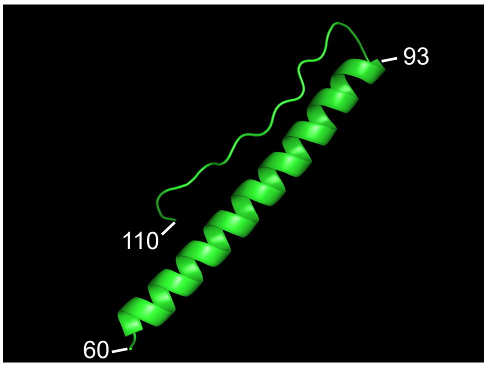

Supplement: Supplementary file 1 — Figure S1. Correlation analysis of strand-specific RNA-Seq and small RNA-Seq. Figure S2. GO analysis of up-regulated and down-regulated genes under nitrogen and phosphate starvation in rice. Figure S3. RNA-Seq read distribution of putative lncRNAs responsive to nitrogen starvation and other stressors. Figure S4. Expression patterns of N-responsive putative lncRNAs and quantitative PCR validation results. Figure S5. Predictions of miR169-targeted rice NF-YAs and 5′ RACE results. Figure S6. Degradome sequencing analysis of genes targeted by rice microRNAs. Figure S7. RNA-Seq read distribution on the genomic region of ammonium transporters, and predicted secondary structures. Figure S8. RNA-Seq and Small RNA-Seq read distribution on the genomic region of AMT2.1 and AMT3.3. Figure S9. Sequence alignment of Chr04G0017 genomic region in rice subspecies. Blue characters indicate predicted open reading frame. Figure S10. Peptide modeling results of Chr04G0017-encoding open reading frame. (PDF 33992 kb) [file 12864_2018_4897_MOESM1_ESM.pdf]
